# Dietary Phytoestrogens and Their Metabolites as Epigenetic Modulators with Impact on Human Health

**DOI:** 10.3390/antiox10121893

**Published:** 2021-11-26

**Authors:** Victor Stefan Ionescu, Alexandra Popa, Andrei Alexandru, Emilia Manole, Mihaela Neagu, Sevinci Pop

**Affiliations:** 1 Cell Biology Laboratory, “Victor Babes” National Institute of Pathology, 050096 Bucharest, Romania; victor.ionescu@ivb.ro (V.S.I.); alexandra.gruianu@gmail.com (A.P.); emimano@gmail.com (E.M.); 2 Faculty of Medicine, “Carol Davila” University of Medicine and Pharmacy, 050047 Bucharest, Romania; andrei.alexandru@stud.umfcd.ro; 3 SC Hofigal Export-Import SA, 042124 Bucharest, Romania; mihaela.neagu@hofigal.eu

**Keywords:** phytoestrogens, epigenome, gut metabolites, microbiota, S-equol, icariin, arctigenin, enterolignans, resveratrol, dietary phytochemicals

## Abstract

The impact of dietary phytoestrogens on human health has been a topic of continuous debate since their discovery. Nowadays, based on their presumptive beneficial effects, the amount of phytoestrogens consumed in the daily diet has increased considerably worldwide. Thus, there is a growing need for scientific data regarding their mode of action in the human body. Recently, new insights of phytoestrogens’ bioavailability and metabolism have demonstrated an inter-and intra-population heterogeneity of final metabolites’ production. In addition, the phytoestrogens may have the ability to modulate epigenetic mechanisms that control gene expression. This review highlights the complexity and particularity of the metabolism of each class of phytoestrogens, pointing out the diversity of their bioactive gut metabolites. Futhermore, it presents emerging scientific data which suggest that, among well-known genistein and resveratrol, other phytoestrogens and their gut metabolites can act as epigenetic modulators with a possible impact on human health. The interconnection of dietary phytoestrogens’ consumption with gut microbiota composition, epigenome and related preventive mechanisms is discussed. The current challenges and future perspectives in designing relevant research directions to explore the potential health benefits of dietary phytoestrogens are also explored.

## 1. Introduction

In the past decades, the research regarding the beneficial or adverse effects of phytoestrogens present in the human diet has intensified, due to their estrogenic or anti-estrogenic potential in humans and animals [1,2,3]. Phytoestrogens are found in a wide variety of foods, including soy-based products, fruits, vegetables and dairy products [4,5,6]. A huge list of health benefits, including a reduced risk of osteoporosis, hormone-dependent cancers, cardiovascular diseases and brain disorders, as well as decreased menopause symptoms, is often ascribed to phytoestrogens. However, they can act as endocrine disruptors, being able to induce adverse health effects, as well [7,8]. Based on their presumptive beneficial effects on human health, the amount of phytoestrogens consumed in the daily diet has increased considerably worldwide [9,10]. Nowadays, a plethora of dietary supplements based on phytoestrogens has overflowed the market, and their consumption has reached high levels, especially within the female population. Thus, there is a growing need for relevant scientific data regarding their impact on human health.

Indeed, new insights of phytoestrogens’ bioavailability and metabolism have been unveiled, and a more complex image of biological activities of absorbed phytoestrogens and their metabolites has emerged [11,12,13]. The phytoestrogens can be active in the human body as free molecules or as gastrointestinal tract (gut) metabolites, being able to interfere with the endogenous estrogen signaling and associated cellular processes [2].

In general, the key factors that are affecting the bioavailability of phytoestrogens are the age and gender of individuals, food matrices, dose frequency and the ADME (absorption, tissue distribution, metabolism and excretion process) properties. Each class of dietary phytoestrogens (e.g., isoflavones, coumestans, prenylflavonoids, lignans and stilbenes) has its own structural particularities, and studies regarding their bioavailability and metabolism are still far from being completed. An important inter-and intra-population heterogeneity of final metabolites production have been observed in human population [14,15]. Their metabolism is mediated both by tissue enzymes and gut microbiota, either prior to absorption or during enterohepatic circulation [16,17]. Only a small percentage (5–10%) of ingested phytoestrogens can reach the small intestine and are available for absorption into enterocytes and then enters into systematic circulation towards target tissues [18,19]. A substantial part of them undergoes extensive metabolization across the liver, small and large intestines, where they are transformed into metabolites with various chemical structures and bioactivities [12,20,21]. The beneficial effects of phytoestrogens on human health are now considered to be partially influenced by their metabolites, such as S-equol, O-demethylangolensin (*O*-DMA), enterolignans and stilbenes derivatives. In some cases, these metabolites have greater biological activities and sometimes have different impacts on targeted tissues than their precursors [12,21].

On the one hand, the composition of gut microbiota can influence the metabolism of phytoestrogens; on the other hand, phytoestrogens and their metabolites can modulate and reshape the gut microbial composition [13]. Understanding their reciprocal influence, and by deciphering the molecular basis of phytoestrogens and microbiome interaction, it can be the key to elucidate their influence on human health.

For many decades, epidemiologic studies have been trying to find associations between dietary components and disease risks. Nevertheless, multiple factors, such as variations in daily consumption patterns of individuals, results based on empirical analyses on predetermined sets of dietary components and human genetics and metabolism heterogeneity have led to inconsistent results. Despite strong preclinical evidence, the association of dietary phytoestrogens with human disease risks has not yet clearly demonstrated [1,7]. Recently, the European Food Safety Authority has indicated that isoflavones intake of 35–150 mg/day is safe and that there is no concern of possible adverse effects in peri-and post-menopausal women [22]. Moreover, new reports have concluded that consumption of phytoestrogens and its correlation with circulating metabolites is not reliable because of high inter-individual variabilities of microbiota composition and genetic polymorphism of phase-I and -II enzymes [12,23].

In recent years, emerging evidence has highlighted the role of dietary bioactive compounds in modifying the epigenome by directly or indirectly engaging in epigenetic mechanisms controling gene expression [24]. Epigenetic mechanisms, such as DNA methylation, histone post-translational modifications, chromatin remodeling and noncoding RNAs expression, represent the link between genotype and phenotype. Each epigenetic mechanism is reversible and is controlled by specific protein classes which attach, dislodge or maintain specific chemical groups that can signal the initiation or inhibition of gene transcription [25]. These proteins, along with chemical marks attached to DNA, RNA and histones, represent the epigenome, a complex regulatory network that modulates chromatin structure and genome function [26]. When this regulatory circuit is discontinued, normal physiological functions are affected, leading to carcinogenesis or occurrence of other chronic diseases [27]. The epigenome is a dynamic network that undergoes continuously modifications in space and time, stimulated by internal and external factors [26,28]. Environmental factors, including diet, can remodel the epigenome during lifespan from embryonic stage until aging in a beneficial or detrimental way. Many dietary components display several biological activities, including antioxidant, anti-inflammatory and anticancer properties that might play a significant role in chronic disease prevention [24]. Importantly, strong scientific evidence implies that consumption of dietary phytochemicals can maintain the epigenome at normal parameters that support a healthy phenotype, and also it can reverse abnormal gene expression [24,29]. For example, it has been demonstrated that dietary phytochemicals can influence the DNA methylation patterns by altering the substrates and cofactors of 5-Methylcytosine (5mC) reaction, or by inhibiting the enzymes of one-carbon metabolism and by blocking the proteins involved in DNA methylation/demethylation activity [29,30].

The present review discusses the new insights regarding dietary phytoestrogens’ bioavailability and metabolism, pointing out the diversity of their gut metabolites and the complexity of metabolic pathways, followed by each class of phytoestrogens (e.g., isoflavones, coumestans, prenylflavonoids, lignans and stilbenes). In addition, it draws attention to emerging scientific data that sustain the epigenetic modulator capacity of each class of dietary phytoestrogens and their metabolites and the generated impact on several cellular and molecular processes connected with human health. The interconnection of dietary phytoestrogens with gut microbiota, epigenome and related oxidative stress events is presented. In-depth studies of dietary phytoestrogens and their metabolites mechanisms of action at the molecular level can represent an effective approach to undestand how to reverse aberrant epigenetic modifications, reshape gut microbiota and suspend abnormal cellular functions, which consequently will prevent and/or attenuate chronic diseases.

## 2. Phytoestrogens—General Data

**Figure 1:**
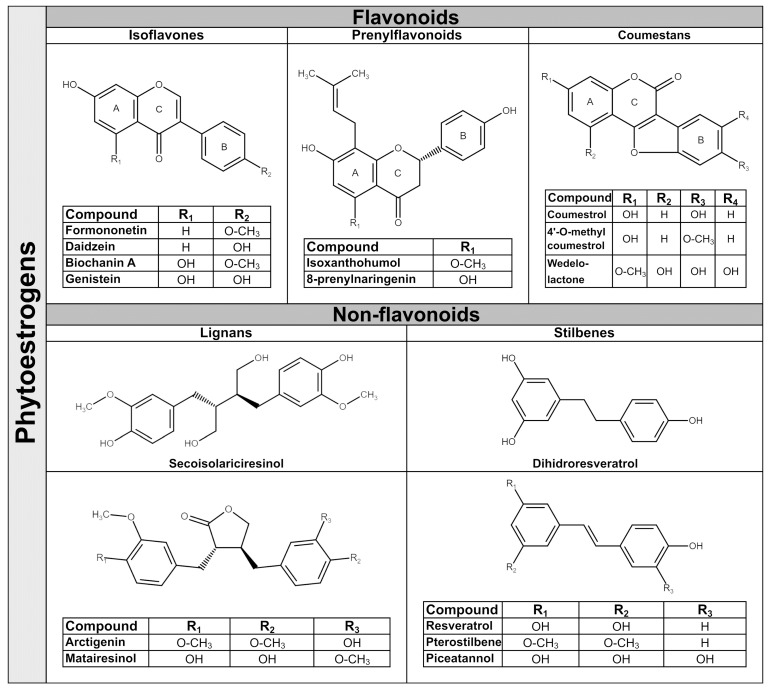
Chemical structure of representative dietary phytoestrogens.

Phytoestrogens are synthesized in plants as secondary metabolites during stress-cultivation conditions and UV radiation, and as a response to pathogens attack. They have antibacterial, antifungal, antiviral and antioxidant properties in plants [31]. The amounts of phytoestrogens produced by a plant increases significantly during extreme growing conditions [32], and by growing the plant in an organic environment [33]. In general, a single plant often contains more than one class of phytoestrogens. For example, the soy bean is rich in isoflavones, whereas the soy sprout is a potent source of coumestrol, the main representative of coumestans class [34]. In terms of chemical structure, phytoestrogens are nonsteroidal polyphenols having several common characteristics with mammalian hormone, estradiol. The structural prerequisite for phytoestrogen molecule to bind the estrogen receptor is the presence of phenolic rings and a pair of hydroxyl groups separated with a similar distance, as in the case of estradiol molecule [35]. The phytoestrogens have been categorized in flavonoids and non-flavonoids. Flavonoids consist of a fifteen-carbon skeleton organized in two benzene rings (A and B) linked by a heterocyclic pyran structure (C) as C6–C3–C6, as Figure 1 shows. The basic flavonoid skeleton can have numerous substituents, including hydroxyl groups usually present at the 4′, 5′ and 7 positions. Most of plant flavonoids have sugar molecules attached to their aglycones, so they mainly exist as glycosides [36]. Further subclassification of flavonoids in isoflavones, coumestans and prenylflavonoids is based on structural differences in the connection between the B and C rings, as well as the degrees of saturation, oxidation and hydroxylation of the C ring [36]. Non-flavonoid phytoestrogen’s structure consists of phenolic acids in either C6–C1 (benzoic acid) or C6–C3 (cinnamic acid) conformations, and are represented mainly by lignans and stilbenes [36].

### 2.1. Isoflavones 

Isoflavones are the main subgroup of plant flavonoids that is found in the *Leguminosae* family, including soy (*Glycine max* L.), red clover (*Trifolium pratense* L.), alfalfa (*Medicago sativa* L.) and species of *Genista* [37]. They have diphenol structures and are produced in higher plant through the phenylpropanoid pathway. Two of most important isoflavones have similar structures; thus, daidzein differs from genistein by lacking a hydroxyl group at C5 position [38]. The representative dietary phytoestrogens’ chemical structures are presented in Figure 1.

Isoflavones are found often in plants as glycosides (genistin, daidzin and glycitin) and in a lower amount as aglycones (genistein, daidzein, glycitein), or as 4′ methylated derivative aglycones (biochanin A and formononetin) [36,39]. The presence of hydroxyl groups and sugars increases their solubility in water, whilst methyl groups confer to them lipophilicity [40]. In the human diet, the main source of isoflavones are soy and soy-derived products, but small quantities of isoflavones are also found in chickpeas, beans, fruits, vegetables and nuts [6,41]. In Western countries, the cow’s milk and dairy products contain significant amounts of isoflavones [5]. Due to their pleiotropic activities, isoflavones are considered as a natural alternative for the treatment of estrogen decrease-related conditions during menopause, cardiovascular diseases and other hormone disorders [42].

### 2.2. Prenylflavonoids

Prenylflavonoids structure contains a flavonoid skeleton that has attached at position 8 of A ring a lipophilic prenyl chain [43]. Prenyl chains appear in various forms, and the most notable are 3,3-dimethylallyl substituent, geranyl, 1,1-dimethylallyl and their moieties. The presence of hydrophobic prenyl radical increases prenylflavonoids cellular uptake and biological functions by accelerating interactions with the phospholipid layers of cellular membranes or hydrophobic target proteins. Therefore, they are considered to be more biologically active than corresponding flavonoids. Prenylflavonoids have a narrow distribution in plants in several families, including Families of *Leguminosae* (*Glycyrrhiza glabra*), *Cannabaceae* (*Humulus lupulus* L.), *Berberidaceae* (*Epimedium brevicornum* M.), *Rutaceae* and *Moraceae* [44]. The most studied prenylflavonoids are those found in hops (*Humulus lupulus* L.), the main raw material for beer production. The representative hop’s prenylflavonoids are xanthohumol (XN); isoxanthohumol (IX), which is produced during the brewing process from XN; 6-prenylnaringenin (6-PN); and 8-prenylnaringenin (8-PN) [43]. In particular, 8-PN can be derived from desmethylxanthohumol (DMX) in the brew kettle, yet it can be converted from IX by human microbiota and by liver enzymes [45]. Other prenylflavonoids which have aroused interest in recent years are icariin, a prenylated flavonol glycoside present in *Herba epimedii* that has been used in Chinese traditional medicine for centuries [46]; and glabridin, which is considered a *Glycyrrhiza glabra* species-specific compound. Glabridin is a prenylated isoflavan with a pyran-substitution at the A-ring and with a high content in dried roots of licorice [47]. As hops are used in beer production, so beer is representing the main source of dietary prenylflavonoids, with IX the major hop prenylflavonoid present in human diet up to 3.44 mg/L [48]. Hops extracts are used in traditional medicine as an antifungal and antibacterial remedy, also to treat insomnia or stomach pain. Recently the hop’s phytoestrogens have gained increasing interest due to their stronger biological activities compared with isoflavones [49]. The presence of prenyl chains allows the prenylflavonoid molecules to interact with the hydrophobic pocket of the estrogen receptors based on in silico modeling studies [50]. The 8-PN is a selective phytoestrogen which has a higher affinity for ERα, having only a 70-times-lower affinity compared to 17β-estradiol [48]. Similar to all other phytoestrogens, prenylflavonoids exert also antioxidant and antitumor activities with greater potential than isoflavones or their flavonoids precursors [51].

### 2.3. Coumestans

Coumestans are produced by oxidation of pterocarpan, a precursor of isoflavonoid phytoalexins from plants, and consist of a benzoxazole fused to a chromen-2-one structure [52]. The coumestans are found mainly in *Leguminosae* family, including alfalfa (*Medicago sativa*), red and white clover (*Trifolium pratense* or *repens*) and soybean (*Glycine max*) [34]. The most-documented coumestans is coumestrol, which is abundant in all species mentioned above. Coumestrol, in addition to flavonoid structure, has a furan ring in the junction between the C and B rings and hydroxyl groups at the C4 and C7 carbons, similar to the structure of estradiol [53]. Interestingly, the coumestrol can be produced in plants from daidzein under stresses conditions such as germination, fungal infection, or chemical elicitors [54]. Other coumestans present in food or medicinal plants are 4′-methoxycoumestrol, repensol, wedelolactone and their derivates [52]. Wedelolactone, the active ingredient of herbal medicine derived from *Asteraceae* family, has been extensively used in South American native medicine as snake antivenom [55]. In traditional Chinese medicine, coumestans are used to treat septic shock and in Indian Ayurvedic medicine as a treatment for liver diseases, skin disorders and viral infections [56]. Coumestrol is considered the most potent phytoestrogen with an affinity for mammalian estrogen receptors only 10–20 times lower than 17β-estradiol [53]. In addition, its antioxidant activity is considerably higher than genistein and daidzein [57]. Coumestans are less common in human diet than isoflavones, but they are present in food plants, including split peas, pinto and lima beans, spinach, broccoli, brussels and soybean sprouts with amounts between 0.025 and 281 mg/kg fw [4].

### 2.4. Lignans

Lignans have a wide distribution in plants, being present in more than 55 plant families, including *Lauraceae* family, especially genera of *Machilus*, *Ocotea* and *Nectandra*; and others such as *Annonaceae*, *Orchidaceae*, *Berberidaceae* and *Schisandraceae* [58]. They are found throughout the plant tissue, namely in roots, rhizomes, fruit, stems, leaves and seeds, with the highest concentrations found in flaxseed [58]. Their biosynthesis originates from the metabolism of phenylalanine with the production of monolignol, the lignan and lignin precursor. Even though lignans are not considered to be dietary fiber, by sharing the same precursor with lignin, an insoluble fiber present in all plant cell walls, can influence lignans’ metabolization [59]. Structurally, lignans are stereospecific dimers of monolignol interconnected between the C8 and C8′ positions, and further linked to either, lactone or carbon bonds. They possess a large structural diversity and are present in plants as aglycones, glycosides with one or more sugar groups, esterified glycosides or as bio-oligomers [60]. Pinoresinol (PINO) is the precursor of the most abundant plant lignans secoisolariciresinol (SECO) and of matairesinol (MAT), which is a dibenzylbutyrolactone. SECO has a dibenzylbutane structure and its diglucoside form, Secoisolariciresinol diglucoside (SDG) accounts for over 95% of the total lignans found in flax [61]. Several other lignans characterize plant foods, including lariciresinol (LARI), medioresinol (in sesame seeds, rye and lemons), syringaresinol (in grains), sesamin and sesamolin (in sesame seeds) [4]. Other lignans, such as arctigenin, have a dibenzylbutyrolactone structure, which is the main component of *Arctium lappa*, being used in Japanese Kampo medicine for its antioxidant, anti-inflammatory and antiviral activity [62]. Plant lignans are known to display a wide range of biological functions, including weak estrogenic and cardioprotective activities, as well as anti-estrogenic and anticarcinogenic properties [17]. In general, plant lignans are considered to be precursors of more bioactive molecules, known as mammalian lignans, enterolactone (ENL) and enterodiol (END), which are produced by colonic microbiota. Plant lignans are the principal source of dietary phytoestrogens of Western diet [63].

### 2.5. Stilbenes

Stilbenes are non-flavonoids containing two phenyl moieties connected by an ethylene bridge that generates two isomers (cis and trans), with trans-isomer as the most stable and biologically active [64]. They are synthetized through the phenylpropanoid-acetate pathway in response of plant’s defense system, as in the case of flavonoids. More than 400 stilbene compounds have been identified in plants, with various structures from monomers to octamers with different substituents, such as glycosyl, hydroxyl, methyl or isopropyl radicals. A high content of stilbenes has been found in species such as *Gnetaceae*, *Pinaceae*, *Cyperaceae*, *Fabaceae*, *Moraceae* and *Vitaceae* [64]. The most studied stilbenes are the monomeric ones, including resveratrol, pterostilbene and piceatannol. They are naturally occurring in fruits, mostly in grapes, berries and peanuts [65]. In general, the occurrence of stilbenes in human diet is limited, but represents an important part of phytoestrogens intake by people consuming a Mediterranean diet or who regularly are drinking wines. Resveratrol is a trans 3,5,4′-trihydroxystilbene and exists mostly as piceid, its glycosidic form, in red and white grape juice [66]. The red-grape juices contain high amounts of trans-piceid, followed by cis-piceid and trans-resveratrol [66]. Piceatannol as a trans 3,4,3′,5′-tetrahydroxystilben that is naturally present in both red and white grapes, berries, passion-fruit seeds and white tea [67]. During the wine fermentation process through hydroxylation, the resveratrol is converted to piceatannol [68]. Pterostilbene is the 3,5-dimethoxy analogue of resveratrol which is found in *Dalbergia* and *Vaccinium* species. The presence of the two methoxy groups makes pterostilbene molecule more liposoluble, increasing its bioavailability as compared to resveratrol [69]. The stilbenes are known for their antibacterial, antioxidant, anti-inflammatory, cardiovascular and neuroprotection properties [70].

## 3. Bioavailability and Metabolism of Dietary Phytoestrogens

The absorption rate of dietary phytoestrogens is determined primarily by their chemical structure and by factors such as molecular size and solubility, extent of glycosylation, hydroxylation, acylation and degree of polymerization [71]. In general, their absorption rate is low, signaling intense metabolism with the formation of metabolites by gut microbiota or by enzymes from liver. Most of ingested phytoestrogens are in glycosidic forms (e.g., isoflavones, lignans and stilbenes), and the first step of their metabolism is their conversion into corresponding aglycones. Metabolism of dietary phytoestrogens in humans follows the detoxification steps of drugs through two phases. Phase I consists of mainly oxidation and hydroxylation reactions catalyzed by enzymes such as cytochrome P450s and flavin-containing monooxygenases [72]. Phase II consists of conjugation reactions, resulting in metabolites with small polar molecules attached that facilitate their excretion in urine or bile [73]. Most of the phase-II metabolites are usually less active or completely inactive than phase I metabolites. Further, the free aglycones and part of gut metabolites can be re-conjugated subsequently by phase-I and -II enzymes within enterocytes and hepatocytes to increase their solubility in body’s fluids [74]. From this reason, there is a high percentage of conjugated metabolites in human plasma. In the case of isoflavones, almost 75% of them are glucuronide conjugates, approximatively 24% are sulfated and only 1% are free aglycones [15]. Once in the bloodstream, phytoestrogens and their metabolites can reach target tissues and, later on, are excreted in urine or bile. Moreover, the metabolites can be de-conjugated by microbiota to release the free aglycones, which are absorbed by the intestine via enterohepatic re-circulation or finally are excreted in feces [20,72]. Bacterial strains from gut are able to catalyze an array of reactions that play key roles in the metabolism of phytoestrogens, including hydrolysis of esterified and conjugated bonds, deglycosylation (removal of sugar moieties), demethylation (substitution of a methyl by a hydroxyl group), dehydroxylation (reduction of hydroxyl groups), dehydrogenation and reduction. In the following subsections, the metabolism of each class of dietary phytoestrogens is presented, pointing out the relevant scientific data gained in the past years.

### 3.1. Isoflavones

Generally, the isoflavones are present in food as glycosides and, to a less extent, as deglycosylated molecules. However, the fermentation process used to obtain specific soy products can increase the concentration of aglycones in processed soy. Once ingested, the glycosidic isoflavones can be hydrolyzed along whole gut by either brush-border enzyme of gut mucosa [75] or by β-glucosidases of different bacterial species, such as *Bifidobacteria*, *Escherichia coli* and *Lactobacillus*. As studies with human subjects have revealed, aglycones are more likely to be absorbed in the small and large intestines, due to their higher lipophilicity and lower molecular weight than the parent glycosides [76,77]. The occurrence of biphasic appearance of isoflavones in plasma, as well as in urine, has been reported [18]. The first peak appears at two hours after isoflavones intake and may represent the rapid transformation of glycosides into aglycones. The second peak appears 6–8 h later and could be accounted for 90% of total isoflavones, corresponding to further biotransformation by gut microbiota of unabsorbed isoflavones [78]. Once the aglycones reached the colon, they can be converted into more or less bioactive metabolites than their precursors. For example, daidzein is hydrogenated to dihydrodaidzein and further converted to *O*-DMA and/or S (−) equol, depending on the presence of specific bacteria strains in the human colon [18]. The S-equol is structurally similar to 17β-estradiol and has a higher estrogenic activity in comparison with daidzein or other isoflavones [79]. In contrast, *O*-DMA is less similar to endogenous estradiol and consequently has a lower estrogenic activity and seems to be less biological active than S-equol or daidzein [80]. Genistein is first reduced to dihydrogenistein, and then to 6′-hydroxy-*O*-DMA (6′-OH-*O*-DMA), which can be degraded to 2-(4-hydroxyphenyl)-propanoic acid [81]. However, it should be noted that one bacterial strain, *Slackia isoflavoniconverten*, can convert genistein to 5-hydroxy-S-equol, a compound that shows a higher antioxidant activity than genistein [82]. Notably, some of isoflavone’s metabolites can be reconverted to aglycones in blood, assisted by efflux transporters. While genistein glucuronide can be re-transformed to genistein, the sulfate conjugates cannot be modified [83]. While the formation of S-equol is well documented, little is known regarding the relevance of the degradation of genistein to 2-(4-hydroxyphenyl)-propanoic acid or to trihydroxybenzene in humans [19]. 

Formononetin and biochanin A can be demethylated by the intestinal microbiota or by hepatic microsomal enzymes to corresponding free aglycones [39]. Moreover, small amounts of their glucuronide and sulfate metabolites with methoxy group at the 4′-position were identified in plasma and bile of animals and in human cells [84]. In addition, in vitro and in vivo studies have demonstrated that the red clover’s isoflavones have different biological activities in comparison with their demethoxylated aglycones [85]. 

Not all human individuals harbor intestinal bacteria that are capable of metabolizing daidzein to biological active S-equol [79], whilst the majority of animal species consuming plants rich in isoflavones can produce S-equol [86]. Observational studies showed that only 30% of the Western population is able to produce S-equol, in comparison with the Asian population, where approximatively 60–70% of individuals are S-equol producers [15,87]. Hitherto, several species of bacteria capable of producing S-equol have been identified, including *Streptococcus intermedius*, *Ruminococcus productus*, *Eggerthella* sp. Julong732, *Adlercreutzia* and *Slackia equolifaciens* [12,88]; however, the abundance of *Asaccharobacter celatus* and *Slackia isoflavoniconvertens* in the individual’s gut microbiota might play a significant role [89]. One study found that a consortium of *Lactobacillus mucosae, Enterococcus faecium* and *Finegoldia magna EPI3 Veillonella* sp. was able to produce S-equol in the presence of colonic fermentation products, such as poorly digestible carbohydrates, but not when fructo-oligosaccharides were added in culture [90]. The *Clostridium* species, which are widespread in human population, are considered to be responsible for isoflavones’ degradation, including daidzein conversion to *O*-DMA [13]. Interestingly, the probiotic *Lactobacilus rhamnosus* JCM 2771 has the capacity to produce genistein from daidzin, affecting the production of S-equol [91]. Human dietary-intervention studies using prebiotics or probiotics in order to increase the S-equol production have shown inconsistent results [87].

S-equol first appearance in plasma is at eight hours after isoflavones ingestion and remains present even 48 hours after intake [77]. In humans, plasma or serum levels of free isoflavones are different depending on the duration and the type of diet. Plasma levels of genistein have been reported to be at 7–18 nM in individuals consuming standard Western diets, with a measurably five-times-higher level in individuals consuming vegetarian diets and for high-soy-diet consumers, reaching hundreds of nanomolars [92]. For example, the serum concentration in Japanese postmenopausal women is, on average, 500 nM for genistein, 250 nM for daidzein and 58 nM for S-equol [93]. Importantly, the apparent isoflavones bioavailability is higher in children than adults, higher in healthy people in comparison with individuals with chronic illness and increased in adults who were exposed to isoflavones rich diet during early periods of life [78]. 

Pharmacokinetics studies of S-equol in animals and humans have proven similar metabolism, including rapid absorption [79]. S-equol has the lowest affinity for serum protein, a high affinity for the estrogen receptors and the highest antioxidant activity of all the isoflavones and their metabolites studied until now. More investigations are needed to characterize the impact of different forms of equol, of racemic equol from commercial nutritive supplements versus intestinal production, as well as the effect of equol conjugates on human health. 

### 3.2. Prenylflavonoids

Hop’s prenylflavonoids have shown a slow to moderate rate of absorption through the intestinal epithelium in animal and human studies [94]. In stomach, chalcone XN can be converted to IX by gastric acid. After that, unaltered XN or IX can reach the small intestine where they accumulate into enterocytes and enter the systemic circulation more slowly than 8-PN [51,94]. In vitro studies have indicated that the phase-II conjugation as glucuronidation and, to lesser extent, sulfation predominates over phase-I metabolism for all tested prenylflavonoids [51]. Moreover, IX can be transformed by liver microsomes to 8-PN at a lower rate, but in the colon this transformation by microbiota is higher, with a conversion efficiency close to 35% [95]. Therefore, the demethylation of IX by the microbiota is the predominant pathway of 8-PN production in humans. The human bacterial strain of *Eubacterium limosum* has been found responsible for 8-PN production. In germ free rats the intestinal administration of this bacterial strain resulted in an increase of up to 80% in 8-PN production after IX ingestion [96]. Notably, *Eubacterium* species are also butyrate producers in humans [97], the butyrate being a metabolite capable to act as inhibitor of histone deacetylases, important proteins part of epigenome [98]. Indeed, the particular strain of *E. limosum* was observed to increase the butyrate production along with 8-PN ones in animal studies [96]. As in the case of daidzein conversion to S-equol, there is an inter-individual variation in humans producing 8-PN from IX. So, individuals can be categorized as poor, moderate and strong producers of 8-PN depending on their phenotypic differences that might affect the pathways of biotransformation of prenylflavonoids [16]. Moreover, the polymorphism of metabolic enzymes can influence the 8-PN biotransformation. For example, enzyme CYP1A2, which is responsible for *O*-demethylation of IX to generate 8-PN, presents a high genetic polymorphism in human population [23]. As a consequence, the plasma concentration of prenylflavonoids and their metabolites varies between individuals. The 8-PN is the most biological active prenylflavonoid, and its production by human microbiota represents an additional contribution to overall phytoestrogens content in humans after beer ingestion. 

The common structure of icariin is 8-prenylkaempferol, with two radicals attached, in which one radical is rhamnose and the other glucose. Removal of rhamnose results in icariside I, while removal of glucose radical produces icariside II. The aglycone form of icariin is icaritin, which can be metabolized into desmethylicaritin by demethylation reaction. In animal models the formation of icariin metabolites depended on the route of administration, icariside II being the main metabolite after oral intake and it is less present if icariin was intravenously administrated [46]. Interestingly, in human studies icariside I was not detected, the only metabolites produced by human bacteria were icariside II, icaritin and desmethylicaritin [99]. Moreover, the pharmacokinetic reports showed a peak of icaritin at 8 h after *Epimedium* extract intake, suggesting that the conversion of icariin to its aglycone takes place primarily at intestinal level [100]. Indeed, under anaerobic conditions the intestinal bacteria (*Streptococus* sp. and *Enterococcus* sp.) transform icariin to icariside II [101], and the *Blautia* sp. is responsible for producing hydrolyzed metabolites, icaritin and desmethylicaritin, which both exhibit estrogenic properties [99]. The tissues distribution has been studied only in animal models. A dependence of gender was observed, with high accumulation of absorbed icariin in liver and lung of male rats, and in females, the accumulation was mainly in uterus [46]. Nevertheless, more investigations should be made in order to clarify the metabolism of *Epimedium* bioactive compounds, the plant being considered to have a strong therapeutic potential for human health.

Glabridin is the prenylated isoflavonoid from licorice which binds to the human ER with about the same affinity as genistein [47]. It is highly unstable under basic conditions and has an inhibitory activity on several human cytochrome P450 enzymes [102]. In reconstituted cytochrome P450 isoforms experiments the glabridin inhibition was a time-, concentration-and NADPH-dependent process, with 50% inhibition at 7 and 12 μM concentration [102]. The inhibition was found to be irreversible through dialysis, and for one isoform the inhibition was associated with the destruction of the heme moiety [102]. Moreover, reports showed that the glabridin is a substrate of the intestinal p-glycoprotein P-gp/MCR1 and this along with hepatic glucuronidation could explain its very low bioavailability compared with other phytoestrogens, even in small rodents (7.5%) [103]. Human studies have showed that a dose of standardized licorice extract up to 1200 mg/day for 4 weeks is safe, and pharmacokinetics of glabridin was linear through all investigated period of time [104]. The inactivation of the major cytochrome P450s by glabridin were supposed to be minimal [104], but the presence of other flavonoids in the licorice extract may additively or synergistically inactivate the phase-I enzymes. Despite several documented studies, the pharmacokinetic parameters of glabridin and its metabolism are far from being elucidate, and further studies will be necessary to better define its bioavailability, the existence of potential bioactive metabolites and the precise profile of its P450 interactions. 

### 3.3. Coumestans

There are no systematic studies on coumestans absorption and metabolism in humans, and the few in vivo studies have reported that coumestrol and wedelolactone have low bioavailability in comparison with genistein and daidzein [105,106]. However, the coumestans could go through an intense metabolic process in the human gut, similar to isoflavones. In rats orally feed with wedelolactone for 3 weeks, approximatively 15–20% of wedelolactone was in unconjugated form and an extensive phase-I metabolism was observed [107]. For coumestrol a maximum concentration of unconjugated molecules was detected 4 h after single oral dose, with approximatively of 70 nM/L in plasma of rats which dropped to 15 nM/L almost 8 h after feeding [105]. Moreover, in vitro experiments have revealed that wedelolactone undergoes glucuronidation, methylation, sulfation and oxidative metabolism after 3 h of incubation with rat hepatocytes [107]. Even that wedelolactone has three phenolic hydroxyl groups attached on its skeleton, glucuronate metabolites were preferentially formed [107]. No specific gut metabolites of coumestans have been reported until now, but the future research probably will bring new insides.

### 3.4. Lignans

The beneficial effect of lignans on human health is stemmed from bioactivities of enterolignans END and ENL which are exclusively produced by the gut microbiota. The END and its oxidation product ENL exert numerous health benefits against breast, colon and prostate cancer, osteoporosis, cardiovascular diseases, hyperlipidemia and menopausal syndrome [108,109]. The complexity and diversity of lignan molecules require a supplemental series of reactions in order to facilitate their absorption in humans, in comparison with flavonoids. In contrast to isoflavones, lignans did not appear in blood immediately after ingestion which suggests a slower rate of absorption and more intense metabolization. Reports based on in vitro experiments, in simulating conditions of the stomach and small intestine, have been showed that lignans such as SDG are resistant to acid hydrolysis [110]. Indeed, the majority of plant lignans suffers marginal alteration during gastric and small intestine passage [111]. However, their deglycosylation may occur via the action of brush border enzymes of small intestine as suggested by the in vivo appearance of SECO in plasma, 5–7 h after the intake of food rich in SDG. Moreover, maximum serum concentrations of END and ENL were attained after 12–24 and 24–36 h, respectively [112]. Therefore, a little amount of aglycones is absorbed in small intestine, with a significant portion of ingested lignans reaching the large intestine for further transformation by the local microbiota [17]. The lignans metabolism has proved to be a multiple-step process catalyzed by a diversity of microbacterial strains. In vitro fermentation experiments with human bacteria species and in vivo studies including dietary interventions in humans have identified a consortium of at least 28 species of bacteria involved in enterolignans production [17,71]. For example, the initial step of SDG metabolism, the deglycosylation can be catalysed by three *Bacteroides* sp. (*B. distasonis*, *B. fragilis* and *B. ovatus*) and two strains of *Clostridium* (*C. cocleatum* and *C. saccharogumia*) [113]. Demethylation of its aglycone requires other bacterial strains, including *Butyribacterium methylotrophicum*, *Eubacterium* (*E. callanderi* and *E. limosum*), *Blautia producta* and *Peptostreptococcus productus* [88,111]. Dehydroxylation of SECO is catalysed by *Clostridium scindens* and *Eggerthella lenta* and the dehydrogenation of END to ENL, and closure of the lactone ring involves bacterial strain of *Lactonifactor longoviformis* [[88,111]. In general, the diglucosides or glycated lignans are following the multi steps metabolism of SDG and for most of them SECO is the intermediate aglycone form. The transformation of arctiin, to its aglycon arctigenin, and then to ENL requires an extra demethylation reaction [17]. Interestingly, the bacteria *Ruminococcus R*. sp. END-1 isolated from human has been able to oxidize enantioselectively (−)-END to (−)-ENL. Moreover, the bacterial strain showed demethylation and deglycosylation activities, and by co-incubation with *Eggerthella* sp. SDG-2 were able to transform arctiin and SDG to (−)-ENL and (+)-END, respectively [114]. Although lignans are widely present in human diet, only few of them can be converted with high efficiency into enterolignans, mainly those with lactone and furan-based structures [115,116]. MAT, LARI and PINO have similar rate of conversion, around 55–65%, in comparison with SDG and SECO which have the highest rate of conversion [115]. In contrast isolariciresinol, also a flaxseed lignan is not converted to either END or ENL [115]. Notably, the bacterial strains capable to generate enterolignans are generally widespread in human population, so no significant inter-population variability has been observed, as in the case of S-equol or 8-PN production. The main factors controlling the plant lignan’s bioactivation in humans are diet, transit time, intestinal redox state and drugs uptake. All these can affect the composition of microbiota and the activities of bacterial strains responsible for enterolignans production [17,106]. Importantly, the microbial dehydrogenation of END to generate ENL is the crucial step in dietary lignans metabolism and the shift toward a major production of ENL is desirable because its stronger association with health benefits [17]. Serum or urinary ENL concentration varies considerably in humans depending mainly on dietary preference and ranges typically between 0.1 and 10 μM [117]. There is a relatively limited information of their tissue distribution and largely it comes from preclinical evaluations of rodent models. In general, all dietary phytoestrogens and their metabolites accumulate in highly perfused tissues such as the liver, intestine, kidney and lung and are present predominantly in their conjugated forms [118]. The preclinical data on mice and rats have revealed that liver contains the majority of the tissue lignans, approximatively 55% of total absorbed lignans. After prolonged exposure to SDG, the concentrations of lignans in skin and kidneys have increased, indicating tissue accumulation. For females, a higher lignan concentrations in heart and thymus has been observed [119]. Moreover, other reports had shown that flaxseed lignans co-administrated with isoflavones can produce more END in plasma than daidzein, and the enterolignans were also present in prostate and breast tissues [119]. Furthermore, clinical studies have demonstrated that the levels of ENL in cancer-free patients are significantly higher than those measured in patients with breast cancer [63,109]. This observation along with other evidence strongly suggests that stable ENL levels can be associated with a reduced risk of hormone-dependent cancers [108,120]. 

### 3.5. Stilbenes

Resveratrol is a lipid-soluble compound with a high cellular membrane permeability, but its low water solubility (< 0.05 mg/mL) affects its oral bioavailability [121]. Even though its systemic bioavailability is low, detectable level of resveratrol in epithelial cells along aerodigestive tract has been observed [122]. At intestinal level, resveratrol can undergo passive diffusion or can bind to membrane transporters [121]. If it is present in bloodstream as free molecule, almost 90% of them can form complexes with albumin and lipoproteins based on in vitro and in vivo experiments and human studies [123]. However, these complexes can be dissociated by cellular receptors of albumin and lipoproteins allowing free resveratrol to pass cellular membranes and so to improve its absorption and tissue distribution. The stilbenes are present in wine and grape juice, mainly as trans-piceid, a glycosidic compound [66]. Whilst trans-resveratrol can passively diffuse the cell membrane, trans-piceid has seen accumulating in cells and tissues to a lesser extent, due to the presence of its sugar radical. Just after passing the brush border membrane, trans-piceid is hydrolyzed by cytosolic or bacterial β-glucosidases releasing trans-resveratrol [124]. An extremely rapid resveratrol conjugation takes place in the intestine and liver, and this intense metabolism seems to be the rate-limiting step of resveratrol’s bioavailability. More than 20 of its derived metabolites have been identified in animals and humans being produced by the major metabolic pathways [125]. The glucuronide and sulphate conjugates from phase-II metabolism are the most abundant [122,125]. Their plasma levels were reported to be higher compared with the ingested resveratrol, according with data from animal and in human studies [21]. The most studied is resveratrol’s reduced derivate, dihydro-resveratrol (DHR), which has a double bond hydrogenated placed between the two phenolic rings. In addition to DHR, two other metabolites have also been identified in human urine: 3,4′-dihydroxy-trans-stilbene and 3,4′-dihydroxybibenzyl (lunularin) [14]. As in the case of the other phytoestrogens there is a large variation between individuals, some are exclusively lunularin or DHR producers, and others are capable to produce both metabolites, and they are called mixed producers [14]. In vitro fermentation experiments have associated the lunularin producers with a higher abundance of *Bacteroidetes*, *Actinobacteria*, *Verrucomicrobia* and *Cyanobacteria* species and individuals with a lower abundance of *Firmicutes* could be either DHR or mixed producers [14]. Two bacterial strains *Slackia equolifaciens* and *Adlercreutzia equolifaciens*, which can produce S-equol from daidzein, have been found to be able to convert trans-resveratrol to DHR [14]. The biological activities of resveratrol metabolites have recently begun to be investigated and DHR seems to be more effective antioxidant than Vitamin E analogue, Trolox [126]. Further studies of resveratrol metabolism and the biological relevance of its metabolites are considered to be crucial for elucidating the mechanism behind resveratrol health benefits. 

Another interesting resveratrol metabolite is piceatannol, which is resulting from hydroxylation reaction catalyzed by phase-I enzymes in liver microsomes and human lymphoblasts [127]. Additionally, piceatannol can be taken directly from diet, being present in high amount in fruits, including grapes, and white tea [67]. Furthermore, following resveratrol administration in mice models the piceatannol was present in high amount as resveratrol metabolite in plasma, skin, and liver tissues [128]. Moreover, after 5 weeks of resveratrol intake, piceatannol was found as a product of phase-I metabolism in the small intestine of mice [129]. The piceatannol is more stable than resveratrol during metabolism probably due to the presence of an additional hydroxyl group located at the 3′-carbon. Furthermore, piceatannol has similar biological effects as resveratrol, and some data have shown that is more potent than its precursor [68]. 

Pterostilbene has a higher bioavailability compared to resveratrol (80% versus 20%) due to the presence of two methoxy groups on its structure, which confers an increased lipophilicity and a better oral absorption [69]. Notably, pterostilbene through phase-II conjugation is transformed mainly in sulfate metabolites [130]. Using human liver microsomes to assess resveratrol and pterostilbene glucuronidation, most pterostilbene (75%) was unchanged in comparison with 32% of resveratrol which remained unconjugated [131]. Pterostilbene has shown suitable pharmacokinetic parameters with no significant toxic effects. Moreover, a high content of pterostilbene has been detected in various tissues, including in brain, proving that it has good blood–brain partition coefficient [69]. Overall, the bioavailability and organs and tissues’ distribution of pterostilbene is higher than resveratrol; thus, it can be considered to be a more bioactive molecule even at a low blood and plasma concentration.

All the aforementioned compounds with existing Chemical Abstracts Services (CAS) numbers are listed in Table S1, available in the Supplementary Materials section.

## 4. The Relationship between Dietary Phytoestrogens and Gut Microbiota: Impact on Human Health

The association between dietary patterns and prevention of disease is probably due to the biological effects (either synergistic or cumulative) of the various components from diet. A number of interrelated biological processes, such as inflammation or immune function, microbiome and metabolites profiles, epigenetic mechanisms, oxidative stress, and metabolic and hormonal responses, have been reported to be modulated by specific diet constituents [2]. The impact of dietary patterns on these biological mechanisms just started to be characterized, and the accumulating evidence suggests that bioactive nutrients can modulate them and consequently can influence human health [2,3,7].

As presented in the previous section, each class of phytoestrogens can be transformed by gut microbiota generating bioactive metabolites and some of them will exert different or stronger biological activities than their parent precursors [85]. In terms of estrogenic capacity of ingested phytoestrogens, their bioconversion may increase the estrogenic potency up to tens or hundreds of times. Regularly, the human diet contains only small amounts of prenylflavonoids, such as 8-PN, but gut microbiota can transform an amount of 4 mg/L of IX (from beer) into 8-PN, resulting in a approximatively 100 times higher exposure of the host to estrogenic metabolites [132]. A diet rich in lignans can expose individuals to up to 75 times to more bioactive metabolites as enterolignans, with potential estrogenic activity [132]. Ingestion of 13.5 g of flaxseed per day for 6 weeks has been reported to lead to micromolar concentrations of ENL and END conjugates in human plasma, being up to 1000–10,000 times higher than the plasma level of the circulating endogenous estrogens [106,116]. Although phytoestrogens are acting as weaker estrogens or anti-estrogenic compounds, their plasma concentrations can be three times higher than endogenous estradiol after daily consumption of two meals based on soy products [133]. In this case, the dietary phytoestrogens more likely may act as endocrine-disrupting agents, inducing negative effects on human health. Therefore, it is important to know the content of phytoestrogens in human diet, and how the phytoestrogens and their metabolites can influence biological processes in human body. In addition to estrogenic or anti-estrogenic activities, phytoestrogens might exert beneficial antioxidant, anti-inflammatory and antitumor activities, yet the knowledge of possible adverse effects induced by the ingested amount are required. Thus, the content of phytoestrogens from our daily meals should be known, and in the next section, we present the latest information about total phytoestrogens amount from some dietary sources.

### 4.1. The Content of Phytoestrogens in Food

More than 300 phytoestrogens have been detected in a large range of legumes, vegetables, fruits and berries, cereals, nuts, alcoholic and non-alcoholic beverage, as well as in processed food products or dairy products [4,5,37]. The phytoestrogens content in raw food varies substantially, being typically as low as a few micrograms per 100 g, yet sometimes can reach levels of hundreds of milligrams per 100 g, as presented in Table 1. It is boteworthy that the reported data are in a broad range mainly because of different methods used for quantification of each class of phytoestrogens. Moreover, as mentioned before the plants are producing phytoestrogens in variable quantities depending on stress conditions and cultivars [31]. Moreover, the data on the content of coumestans, prenylflavonoids and stilbenes in raw or processed food are rather limited, and more information is needed in this regard.

As Table 1 shows, one food source can contain several classes of phytoestrogens, yet other phytochemicals with biologic activities are presumed to be present. Whole soybean can contain large amounts of isoflavones, along with coumestans and lignans [4]. The flax and sesame seeds have the highest concentration of lignans, mainly SDG and sesamin, but also isoflavones and traces of coumestans [134,135]. Notably, relatively large quantities of phytoestrogens have been found in dairy products, mostly microbiota’s metabolites such as S-equol and ENL from cattle feed with red clover and forages rich in lignans [71]. When feeding cattle with red clover, the level of S-equol in milk can range between 15 and 650 g/L; moreover, larger quantities of S-equol were identified in organic milk in comparison with conventionally produced milk [5,6].

An extensive European study has described the phytoestrogens intake and the food sources, highlighting their variability in European diet as depending on regions and lifestyle characteristics [9]. In the Mediterranean diet, which is considered to be the healthiest one, the main contributing dietary phytoestrogens were lignans (58.1–67.3%), isoflavones (30.4–37.9%) and coumestans (1.5–3.3%), followed by enterolactone (0.7–0.8%) and S-equol (0.2–0.3%) from dairy products [9]. In Europe, USA and Canada, the consumption of phytoestrogens comes mainly from tea, coffee, wine, fruits, and vegetables with an average intake of 2 mg/day [19]. Whilst, in Asian populations isoflavones from soy-based food and lignans from green tea are the main source of phytoestrogens, with a daily intake from 16 to 70 mg/day, but sometimes can reach 120 mg/day [42].

As mentioned, the food matrices have strong influence on the absorption of phytoestrogens [81]. They are present mostly in glycosidic forms in raw food, so are being less absorbed in humans [76,112]. Western diet consumers are eating more non-fermented soy products, such as soy milk and tofu containing primarily the glycosidic isoflavones, whilst in traditional Asian diet predominates the fermented soy products with high content of aglycones, which are absorbed rapidly [4]. Moreover, the simple preparation of food can destroy the chemical structures of phytoestrogens and consequently alter their biological activity. For lignans, a high roasting temperature had caused degradation of both aglycone and glycosidic structures of rye and sesame seeds [138]. To conserve the nutritionally content of lignans during production of processed foods, several aspects should be taken into consideration such as their chemical structures, water content and the applied temperatures [138]. Whether mixtures of phytoestrogens present in daily meals at relevant doses can synergize or antagonize with endogenous estrogens is still under debate, yet the important role of the gut microbiota with regard to the bioavailability and bioactivity of phytoestrogens just started to be unveiled [132,139].

### 4.2. Reciprocal Modulation between Dietary Phytoestrogens and Gut Microbiota

The community of microorganisms populating human guts represents microbiota and their collective genomes form the microbiome which encodes a number of genes with more than 100 times larger than human genome [140]. Moreover, the microbiome of two different individuals is highly different compared to their genomic variation; thus, the genome identity in humans is about 99.9% [141], and gut microbiome can be just up to 10–20% identical [142]. Diet composition has a definite role in the taxonomic and functional profile of the microbiota, and it consequently influences the microbiome. Furthermore, many dietary components are metabolized by commensal or symbiotic gut microbes into bioactive molecules that could support cellular mechanisms for disease prevention. Increasing evidence suggests that the gut microbiome can influence chronic disease predisposition and has a definite role for maintaining the well-being of individuals [2]. A plant-based diet can induce the development of diverse and more stable microbial strains with a favorable impact on human health. Phytoestrogens, as well as plant polyphenols, have increased *Bifidobacterium* and *Lactobacillus* population in human guts, which can induce anti-pathogenic and anti-inflammatory effects, along with cardiovascular protection [11]. Interventional studies have also established that changing from a carnivorous diet to a plant-based one is resulting in a gradual decrease in abundance of *Alistipes, Bilophila* and *Bacteroides*, as well as all bile-tolerant symbiotic microorganisms; and an increase in *Firmicutes*, which preferentially metabolizes plant polysaccharides [11]. Importantly, the *Firmicutes* species are responsible for converting trans-resveratrol to lunularin [14]. By assuming a short-term consumption of diets based exclusively on animal or plant products, the microbial community structure can be rapidly altered, and thus the overall microbial gene expression is changed with impact on human health [143]. Therefore, a diet should be balanced, containing different nutritional components able to support the needs of a healthy human body.

The consumption of phytoestrogens can modulate the gut microbiota composition. A variation in microflora species between S-equol and non-S-equol producers has been observed in several human studies [13]. In addition, an association of S-equol production with the quantitative increase of *Faecalibacterium prausnitzii* and *Lactobacillus–Enterococcus,* which are two dominant species of human colonic bacteria, is indicating that some phytoestrogens might selectively modulate intestinal environment through their metabolites [13]. Recent data have shown that a diet enriched in fibers (e.g., flaxseed gums) induces an anti-obesity effect, along with alteration of microflora community in obese rats and mice [144,145]. The lignan based diet has decreased the relative abundance of *Clostridiales*, and enriched the colonic microbiota with species such as *Clostridium* and *Enterobacteriaceae* [144]. The anti-obesity effect in mice has been associated with an increased abundance of the *Clostridia* genus, which is capable of producing a high level of butyrate [145]. The resulting shift in gut microbiota composition has restored the necessary levels of butyrate and lactic acid, leading to the modulation of gene expression of colonic enteroendocrine cells [145]. Lignan-based diets have improved the colon health, and as several in vivo and interventional studies have demonstrated, they have induced the modulation of gut microbial structure and increased the production of short-chain fatty acids (SCFAs) [13]. Similar to lignans, the stilbenes intake can modulate gut microbiota composition, specifically by increasing the ratio of *Bacteroidetes* to *Firmicutes* species, along with decreasing the abundance of *Clostridium* genus and *Lachnospiraceae* family [146]. Along with gut modulation, a decrease of expression of genes involved in fatty acid synthesis, lipogenesis and adipogenesis; improved carbohydrate metabolism; glucose homeostasis; and lower diabetes risk can be achieved [146,147]. However, there are in vivo studies which have reported no impact of resveratrol supplementation on the ratio of *Bacteroidetes* to *Firmicutes* species, and yet the bioactivity of resveratrol on metabolic syndromes has been observed [148].

Moreover, phytoestrogens, as phenolic compounds, may have antimicrobial activity and can interact with the pathogen bacterial strains [149]. Consequently, they might modulate the diversity of the colonic microflora by inhibiting the pathogens, or by increasing the beneficial bacterial populations, thus contributing to the improved health of the individual.

Convincing evidence to support a link between diet and microbiome in cancer prevention comes from studies on dietary patterns with a high intake of fibers. Dietary fibers undergo bacterial fermentation in the colon to yield butyrate, which acts as a histone deacetylase inhibitor and is able to suppress the growth of colorectal cancer cells in vitro [150]. High fiber intake also encourages the growth of bacteria species that can transform the fibers into other SCFAs, such as acetate and propionate, which, along with butyrate, have an impact on human epigenome [151,152]. As mentioned, the *Eubacterium limosum* strain converts IX to 8-PN [95], but it is capable of producing butyrate in humans [97]. The SCFAs have positive health effects, such as improved immunity response, blood–brain barrier integrity, provision of energy substrates, intestine homeostasis and epigenome modulation [11]. Furthermore, the SCFAs can act as endogenous ligands for two group of orphan G-protein-coupled receptors (GPCRs), also known as free fatty acid receptors (FFARs). The discovery of SCFAs’ capacity to bind and activate FFAR2/GPR43 and FFAR3/GPR41 unveiled the multiple ways of which metabolism and immune system are interconnected [153]. These two free fatty acid receptors are expressed in several human cell types related to the immune system, adipose tissues, gut and in pancreatic β-cells [154]. Moreover, FFAR3/GPR41 appears to be highly expressed in the neurons of sympathetic and enteric nervous system, where upon activation by SCFAs accumulating in the intestine, FFAR3/GPR41 can be signaling to the brain via neural circuits to regulate intestinal gluconeogenesis [154]. Functional expression of FFARs in the nervous system implies a possible connection between nutritional status and autonomic nervous system function. These recent findings demonstrate that free fatty acid receptors might mediate the beneficial effects of SCFAs, with an impact on intestinal homeostasis, energy metabolism, immune system function and neuronal signaling.

Furthermore, phytoestrogens have a direct influence on the aryl hydrocarbon receptor (AhR), which is an important factor in intestinal homeostasis [155]. The AhR, known as a ligand-activated transcription factor, is able to integrate microbial and gut metabolites’ signals, along with environmental and dietary stimuli into complex transcriptional networks in a cell-type and context-specific approach [156]. Isoflavones are natural ligands of AhR, with biochanin A and formononetin having the more potent agonist activities [157]. Moreover, prenylated flavonoids, such as icaritin, 6-PN and 8-PN, but not IX, are exhibiting a unique agonist potential in comparison with their parent precursors by selective upregulation of the P450 1A1-mediated estrogen detoxification pathway [158]. Phytoestrogens can produce ligands for AhR, therefore connecting gut lumen environment and cellular processes with the impact on human health by activating cyto-protective and antioxidant responses [159].

**Figure 2:**
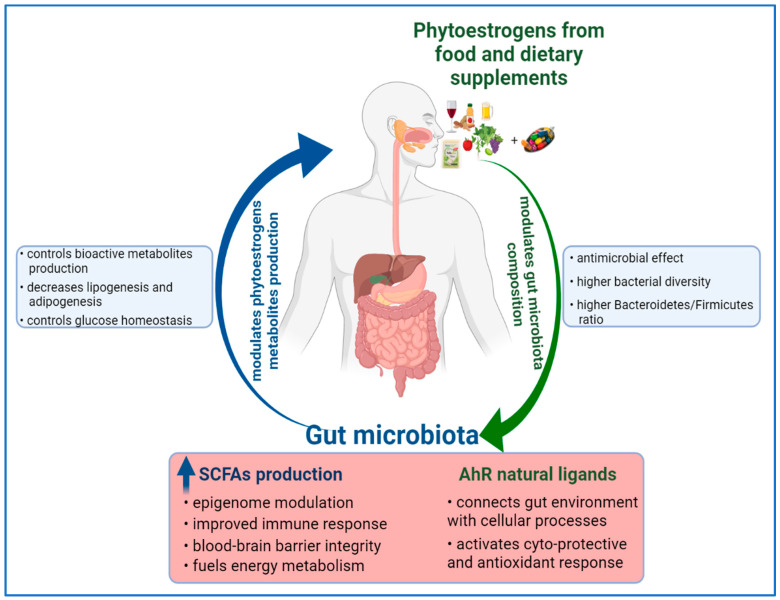
Reciprocal modulation between dietary phytoestrogens and gut microbiota.

In conclusion, the phytoestrogens-based diet might exert health benefits or adverse effects to the host via modulation of gut microbiota. Likewise, the gut microbiota can influence the phytoestrogen metabolites production, with impact on human health, as Figure 2 shows. 

In-depth studies to identify new colonic microbes and their enzymes involved in the metabolism of each class of phytoestrogens are critical for understanding their beneficial or adverse effects on human health. Moreover, the synergistic or cumulative effects that can be induced by different types of phytoestrogens absorbed in human body should be studied, both in terms of modulating the bacterial microflora and their influence on different cellular processes.

## 5. Epigenetic Modulator Capacity of Dietary Phytoestrogens

Dietary phytoestrogens and their metabolites are known for their capacity to interact with estrogen receptors, which are expressed widely in the human cells; however, other important biological activities have been highlighted [7,24,63]. The major mode of action by which phytoestrogens exert their possible health effects is based on their estrogenic or anti-estrogenic activity by binding to estrogen receptors. In Table 2, we present several dietary phytoestrogens which are able to imodulate estrogen-depending signaling pathways.

Resveratrol metabolites could be more efficient antioxidants than their precursors [126], and the prenylflavonoids can induce an antioxidant response much stronger than parent isoflavones [158]. Coumestrol is considered the most potent phytoestrogen, and in addition, its antioxidant activity is considerably higher than genistein and daidzein [57]. Moreover, S-equol has a strong affinity for estrogen receptors and also the highest antioxidant activity among isoflavones [79]. Recently, in a study on adults’ lignans consumption, ENL and END levels from plasma have been inversely associated with markers of chronic inflammation, such as C-reactive protein [174]. The mechanisms underlying the antioxidant and anti-inflammatory effects of phytoestrogens are still being investigated [139], but scientific evidence in recent years interconnected these properties with the potential ability of phytoestrogens to act as epigenetic modulators [7,175]. Epigenetic mechanisms control gene expression, and by modulating one mechanism, phytoestrogens can act on several genes and thus can influence signaling pathways and complex cellular processes. Mainly, the phytoestrogens are affecting the activities of proteins part of epigenome regulatory mechanism, including DNA-methyltransferases (DNMTs), DNA-demethylases (TETs, TDGs), histone deacetylases (HDACs), histone-acetylases (HATs), histone methyltransferases (HMTs) and histone demethylases (HDMs) [25,26,176]. The DNA methylation process is carried out by DNMTs and occurs at CpG islands, which are short DNA sequences located at gene promoters or in noncoding regions scattered throughout the genome. DNMT3A and DNMT3B proteins catalyze the addition of a methyl group to cytosine at five positions (5-mC), whilst DNMT1 maintains the somatic inheritance of DNA methylation during cell division [26]. 

Combinations of histone post-translational modifications are able to signal different modifications on chromatin regions and create transcriptionally activated or repressed gene expression sites [177]. Although the epigenome has not been completely elucidated, histones’ lysine’s acetylation (H3K9ac, H3K14ac and H4K16ac) and trimethylation (H3K4me3) are able to establish transcriptionally active sites; instead, H3K9me3, H3K27me3 and H4K20me1 are signaling the silencing of gene expression [177,178]. The heterochromatin regions can become unpacked when the positive charge of the lysine residues from histone tails are acetylated by HATs. As a consequence, the chromatin accessibility to transcription factors and RNA polymerase II increases, resulting in gene expression. On the other hand, HDACs can restore the compact chromatin by erasing the histone acetylated marks. Deacetylation of a lysine residue from histones may allow its further modification by HMTs, and also intermediate the recruitment of DNMTs for reinforcing the close chromatin status. Together, these epigenetic changes will enable a progressive decrease in the accessibility of DNA to the transcription machinery and hence an increasing transcriptional silencing [25].

Several classes of HDACs are using histones, as well as non-histone proteins as substrate for deacetylation reaction. A particular class of HDACs, the sirtuins family, possesses two enzymatic activities, namely mono-ADP-ribosyltransferase and NAD+ dependent deacetylase, and is involved in the regulation of many cellular processes [179]. One of the most remarkable members of sirtuins family is SIRT1, which can deacetylate both histones and a wide range of non-histone proteins. SIRT1 has a preference to deacetylate the lysine 16 of histone H4 (H4K16ac). This epigenetic mark has been linked to the regulation of cell-cycle progression, active transcription, DNA repair and DNA replication [180]. Moreover, hypoacetylation of H4K16, along with hypomethylation of H4K20, has been proposed as a hallmark of human cancers [181]. By exercising its deacetylation activity, SIRT1 acts as a chromatin modifier, transcriptional regulator and metabolic regulator. Thus, it modulates important cellular functions, such as apoptosis, cell growth, cellular senescence, oxidative stress response, metabolism and tumorigenesis [179,180]. 

Another layer of epigenetic mechanism with an impact on gene expression is represented by small and long non-coding RNAs (microRNAs and lncRNAs) which are able to modulate gene expression without changing DNA sequences. Noncoding RNAs are not translated into proteins, being considered part of the regulatory mechanism of gene expression at both transcriptional and post-transcriptional levels. Notably, the expression of non coding RNAs is under the control of epigenetic regulation mechanisms, including DNA methylation, RNA modification and post-translational modification of histone [182,183]. MicroRNAa (miRNAs) are short RNA sequences with ∼22 nucleotides of lengths, which are acting as epigenetic modulators, by affecting the protein levels of the target mRNAs without modifying the gene sequences [182]. They can bind to target messenger RNAs (mRNAs) through nucleotide complementarity, after which the protein translation is terminated by its mRNA degradation. MiRNAs are considered to be the fine-tuning regulators of protein expression, which can establish the timing and the level of specific protein expression.

The lncRNAs are RNA transcripts longer than 200 nucleotides that are considered to be important regulators of the epigenome. Their molecular mechanism of action depends on cellular localization and the type of interacting molecules; thereby, lncRNAs can be classified as signal, decoy, guides or scaffold [183]. Signal lncRNAs regulate transcriptional activity or signaling initiation, whilst decoy lncRNAs bind and titrate out gene regulatory elements, such as proteins, mRNAs and miRNAs. Nuclear lncRNAs can bind transcription factors or chromatin modifiers proteins (e.g., DNMTs, HATs, HDACs and PcGs), whereas, in the cytoplasm, they function as a sponge to attract proteins and miRNA/RISC complexes away from their targets [183,184]. Acting as scaffolds, lncRNAs can coordinate the formation of distinct epigenetic regulatory complexes on specific chromatin regions, dictating their active or inactive transcriptional status. They have the ability to guide protein complexes to both close and distant genomic loci which allow them to regulate gene expression on a genome-wide scale [184]. In conclusion, as epigenetic modulators, lncRNAs can increase or repress transcriptional activity by controlling the deposition of histone marks and DNA methylation pattern on chromatin regions. The reciprocal interconnection of non-coding RNAs with epigenetic pathways appears to form a feedback loop that have an extensive influence on gene expression throughout the genome. Therefore, any dysregulation may affect physiological and pathological processes and will contribute to a variety of diseases.

Emerging scientific data suggest that a diet rich in vegetables and fruits can significantly reduce the risk of chronic disease development, due to the action of nutrition bioactive constituents, which can regulate the gene expression [176,185]. Remarkably, many phytochemicals, including phytoestrogens, may act through epigenetic mechanisms, such as modulation of DNA methyltransferases (DNMTs), histone deacetylases (HDACs) activities and non-coding RNA expression [24,30,176]. In the next sections, the epigenetic modulator capacity of phytoestrogens and of their metabolites is presented, underlining their mode of action with effect on different biological processes. 

### 5.1. Isoflavones

Isoflavones have been intensely studied in connection with their epigenetic modulator potential in chronic diseases, especially in cancers. Genistein is acting as a chemotherapeutic agent in various cancer cells, modulating cell proliferation, apoptosis and metastasis [186]. Indeed, anticancer properties of genistein have been associated with re-expression of tumor-suppressor genes that were methylation-silenced during carcinogenesis [175,187]. For instance, genistein reversed hypermethylation of ATM, APC, BRCA1 and PTEN tumor-suppressor genes and restored the ER expression in ER positive and triple-negative breast cancer cells by inhibiting the DNMTs’ activities [188,189]. In general, the BRCA1 and BRCA2 mutations imply a high risk of breast and ovarian cancers, yet their epigenetically silenced promoters are found frequently in many types of cancers [189]. Moreover, in the case of ER positive cells, genistein epigenetically had re-expressed BRCA1, but also acted as antagonist of AhR [190]. In addition, genistein and daidzein treatment of breast cancer cells decreased the expression levels of MeCP2 protein [191], which have a methyl-CpG-binding (MBD) domain that recognizes and binds to 5-mC regions of DNA. The daidzein gut metabolite, S-equol, at physiological concentration of 2 µM and after long-term treatment, was acting as demethylation agent of BRCA1 and 2 promoters in breast cancer cells but not in normal mammary gland cell line [192]. In general, isoflavones have shown DNA demethylating capacity, with genistein as the most potent among tested isoflavones, by reducing the promoter methylation of genes involved in preventing cancer development [187,190,191,193,194].

The epigenetic modulation capacity of isoflavones has been observed in correlation with histone posttranslational modifications. Thus, genistein treatment can affect different epigenome modifier proteins, including histone deacetylase (HDACs) and histone H3 Lys 9 methyltransferase (HMT) by reducing their enzymatic activities in cervical cancer cells [194]. The two most studies isoflavones (genistein and daidzein) and the metabolite S-equol have been reported to demethylate transcriptional repression marks, such as H3K9me3 and H3K27me3, concomitant with modulating the EZH2 protein expression, which is controlling and maintaining the repressed chromatin status in breast cancer cells [195]. Moreover, epigenetic marks, such as H3K4ac and H4K8ac, signaling the active chromatin, became hyper-acetylated, accompanied by increased expression of transcriptional activators P300 and SRC3 [195]. Another study reported that genistein alone or in combination with trichostatin A can reactivate ERα expression in the ER negative cells. An enhancement of ERα expression and increased global H3K9, H3 and H4 acetylation, along with decreased DNMT1 expression and HDAC activity, were observed [196]. Furthermore, mice exposed to a long-term genistein diet resulted in having a high expression of ERα in spontaneous breast tumors, preventing the occurrence of a more aggressive ER-negative type of breast cancer [196]. The protective effect of genistein can be assumed to be at least in part due to its epigenetic modulator capacity, since HDACs activity was significantly reduced in mice breast tumors [196]. Studies on genistein treatment of precancerous and fully developed breast cancer cells have shown an increased expression of tumor-suppressor genes p16 and p21 through enrichment of transcriptional active markers, such as acetyl-H3, acetyl-H4 and H3K4me3 [188]. Moreover, genistein induced a stronger anti-proliferative effect and pro-apoptotic response in precancerous cells than in breast cancer cells [188], which might suggest that its anticancer properties could be more effective at an early stage of tumorigenesis. In addition, the genistein-enriched diet has prevented tumorigenesis and inhibited cancer development in breast-cancer mice xenografts [188]. Isoflavones can modulate miRNAs expression with impact on their anticancer activity. In vitro studies have demonstrated that onco-miRNAs expression, such as miR-155, can be significantly reduced by treating breast cancer cells with physiological concentration of 1–10 µM genistein [197]. Conversely, the expression levels of miR-155 targets were upregulated in response to genistein treatment, including pro-apoptotic and anti-proliferative proteins: FOXO3, PTEN, casein kinase and p27 [197]. Recently, Lynch et al. have demonstrated that the long-term treatment of PC3 prostate cancer cell line with 40 µM genistein has induced hypomethylation of miR-200c gene/cluster resulting in increased expression of miR-200c [198]. Furthermore, genistein can modulate the expression of tumor-suppressor Smad4 in prostate cancer cell lines via multiple epigenetic mechanisms. Thus, genistein can downregulate miR-1260b that targets Smad4, or genistein can induce DNA methylation and histone modifications at Smad4 gene promoter in prostate and renal cancer cells [199,200].

There are a few studies exploring the possible connection of biochanin A and formononetin with epigenetic mechanisms. Notably, biochanin A effectively and reversible inhibited the Lysine-specific histone demethylase 1A (LSD1) activity in vitro, in gastric cancer cells at physiological concentration [201]. However, emerging evidence is suggesting that red clover isoflavones might have the ability to modulate miRNAs expression in different types of cancer cell lines [202]. Whilst biochanin A seems to promote ERα-positive cells’ proliferation through miR-375 activation [203], the formononetin at its physiologic concentration showed the same pro-proliferative effect and anti-apoptosis activity via upregulation of miR-375 in aortic endothelial cells, but not in breast cancer cells [204]. Moreover, in ovariectomized rats, formononetin intake have increased uterine weight and induced the upregulation of miR-375, ERα and Bcl-2 and the downregulated RASD1, but no effect was observed on mice bearing MCF-7 xenografts [204]. The formononetin ability to trigger selectively the miR-375/RASD1/ERα pathway may suggest that its long-term use can lower the risk of postmenopausal breast cancer development. Formononetin can modulate in a time-and dose-dependent manner the miR-21 expression in bladder cancer in vitro by significantly inhibiting cellular proliferation and invasiveness and inducing apoptosis. In addition, reduced expression of miR-21 was followed by increase of PTEN, and downregulation of p-Akt [205]. Moreover, formononetin-combined therapy may enhance the therapeutic efficacy of doxorubicin in glioma cells by preventing EMT through inhibition of HDAC5 [206]. In vivo experiments on rats with type 2 diabetes the formononetin long-term treatment has been able to control hyperglycemia and insulin resistance and also to reduce oxidative stress through SIRT1 re-expression in kidney tissues of diabetic animals [207]. Moreover, a recent study has demonstrated that formononetin can attenuated triple negative breast cancer cells malignancy by suppressing the lncRNA AFAP1-AS1 expression with an influence on the miR-545-3p/CDK4 and miR-195/Raf-1 axes, which are associated with triple-negative breast cancer chemoresistance [208]. Recently, it has been demonstrated that S-equol is involved in miRNAs’ expression regulation as exerting its antitumor activities [209]. S-equol has induced apoptosis in MCF-7 cells by modulating the PI3K/AKT pathway through the upregulation of miR-10a-5p expression in a time-and dose-dependent manner [209]. Interestingly, a new report has shown that isoflavones, particularly genistein, can reduce the interaction of an oncogenic lincRNA HOTAIR with chromatin remodeling factors, resulting in renal tumor cell line malignancy suppression [210]. Thus, new approaches to investigate the epigenetic modulator capacity of isoflavones at lncRNAs level could be initiated. Other examples are presented in Table 3.

In vivo studies have demonstrated the direct effect of isoflavones on the epigenome. The exposure of newborn rats to high doses of coumestrol and equol caused hypermethylation and subsequent inhibition of the proto-oncogene H-ras independent of newborn gender [227]. Furthermore, supplementing the maternal diet with genistein was reported to alter loci-specific DNA methylation status in the offspring of transgenerational Agouti mice [220]. Moreover, the genistein-induced DNA hypermethylation persisted into adulthood, decreasing ectopic Agouti expression and protecting mice from developing obesity. Thus, this study provided direct evidence that in utero dietary genistein affects gene expression by permanently altering the epigenome [220,287]. Genistein may have cancer suppressive effect through modulating early epigenetic events related to carcinogenesis. Early exposure to xenoestrogen chemical Bisphenol A may predispose humans to develop precancerous lesions by altering DNA and histone methylation pattern and dysregulated miRNAs expression [288]. Bisphenol A is used in plastic industry and has a negative impact on the epigenome leading to multiple dysregulations of normal development [289]. The simultaneous exposure of pre-pubertal rats to Bisphenol A and genistein significantly reduced the altered DNA modifications. Moreover, HPSE and RPS9 genes which have a strong predictive value for long-term survival of breast cancer patients have been identified to be hypomethylated and highly expressed in mammary glands of exposed rats [290]. A recent report of Shanghai Breast Cancer Survival has been established that long-term soy-based diets might lead to increase expression of tumor-suppressor miRNAs involved in TP53 cancer-related network and decreased expression of oncogenes such as KRAS and FGFR4 [291].

The investigations of possible mechanism of isoflavones epigenome modulator activity have revealed that genistein can fit and bind to catalytic domain of DNMT1, but not to DNMT3A and B [189]. Indeed, genistein showed a strong inhibitory activity against DNMT1, but not to other DNMTs, in several tumor cell lines [189,194]. However, it appears that similarly to other bioactive compounds, isoflavones act differentially to impact DNA methylation states, depending on the gene-loci or gene function. An additional level of complexity for the epigenetic effects of genistein concerns the dose-dependent effects [186]. Interestingly, the changes in miRNAs expression induced by isoflavones are similar to those obtained by demethylating agent 5-azacytidine in prostate cancer experiments [198,292]. This might suggest that the regulatory impact of isoflavones on miRNAs expression could be related to their ability to inhibit DNMTs activities such as 5-azacytidine, and accordingly the promoter methylation status of the epigenetically suppressed miRNAs is reversed. Indeed, the decrease of 5-mC marks at promoters of miR-29a and miR-1256 after genistein treatment of prostate cancer cells had been observed. The re-expression of two miRNAs significantly downregulated their direct targets, TRIM68 and PGK-1, leading to the inhibition of prostate cancer cells growth and invasion [292].

### 5.2. Prenylflavonoids

Recently, the epigenetic modulator capacities of prenylflavonoids have started to be studied based on assumption that many of their biological activities could be related with epigenetic mechanism. Prenylflavonoids, especially those derived from hops are known as estrogen-synthesis modulators, but can also affect the estrogen metabolism through the AhR pathway [293]. The 6-PN has demonstrated to be an AhR agonist by targeting ER for proteasomal degradation, which causes CYP1A1 transcription but not CYP1B1 [164]. The ERα-mediated regulations of selective transcription of AhR-mediated genes can be controlled by epigenetic mechanisms such as DNA methylation. Indeed, 6-PN and a standardized hop extract selectively induced upregulation of AhR-dependent estrogen detoxification pathway by reducing of DNMT1-mediated inhibition of CYP1A1 transcription [228]. Moreover, Venturelli et al. [229] have shown by in silico studies that both 6-PN and 8-PN can fit into catalytic site of HDAC 2, 4, 7 and 8 enzymes and interact with Zn ion for catalytic activities, so may be acting as pan-HDACs inhibitors. The in vitro experiments revealed that 6-PN and 8-PN can induce a rapid hyperacetylation of H3 histone within few hours after applying the PFs treatment on melanoma cells. Their strong HDACi activity have been correlated with an anti-proliferative effect and prolong treatment with prenylflavonoids resulted into G2 cell-cycle arrest and necrosis. These results have suggested that cells death may be induced by 6-PN and 8-PN via the interplay of inhibition of HDACs activities and Phospho-S6 Ribosomal Protein decreased expression by downregulation of pERK/pP90 pathways [229].

Icariin might act as epigenetic modulator of SIRT6 in several cell and animal model diseases, such as breast cancer [233], leukaemia [237], inflammation and aging [232,234]. In all studies icariin has shown inhibitory activity against NF-κB signaling pathway through SIRT6-mediated deacetylation of H3K9. As a consequence, several NF-κB target genes involved in migration and invasion of breast cancer cells were inhibited. The N-cadherin and MMP-2 protein expression levels were significantly downregulated by icariin treatment [233]. The SIRT6 protein is a deacetylase of histone H3K9 and by its involvement in epigenetic mechanism is able to regulate several fundamental processes such as DNA repair, gene expression, telomeric maintenance and genomic stability [294]. Moreover, SIRT6, similar to its partner SIRT1, as a member of HDACs class III, is capable to facilitate the cellular stress response and could regulate glucose and fat metabolism, thereby inhibiting the inflammatory response [232]. The activation of SIRT1, followed by deacetylation of H4 histone has been observed in paclitaxel-induced neuropathic pain rats [236], proving that icariin, as with other phytoestrogens, can modulate the sirtuins deacetylation activity.

Reports have suggested that icariin inhibited the PI3K/AKT and MEK/ERK signaling pathways through regulation of miR-625-3p, and consequently affected cell proliferation, apoptosis, migration and invasion of thyroid cancer cells [295]. By regulation of miR-21 expression, icariin inhibited proliferation and induced apoptosis and increased expression of tumor suppressors PTEN and RECK in ovarian cancer cells [235].

Furthermore, icariin can restore the ABCB1 expression through promoter demethylation in human mesenchymal stem cells (hMSCs), extracted from patients with osteonecrosis [231]. The ABCB1 gene encodes P-glycoprotein, a drug transporter protein that determines the uptake and efflux of drugs in cells [296]. The icariin treatment restored the dynamic balance between osteogenic and adipogenic differentiation of hMSCs, increased P-glycoprotein expression and decreased oxidative stress [231].

Data from the recent literature have suggested that glabridin modulates expression of miR-148a through DNA demethylation in breast cancer cell lines [[167,230]. Glabridin decreased the average methylation level, reducing DNMT1 and DNMT3 expression in the promoter regions of miR-148a, enhancing its expression levels. Consequently, miR-148a suppresses the activation of TGF-β/SMAD2 signaling pathway, and attenuates cancer stem-cell-like functions in hepatocellular carcinoma and breast cancer cells in vitro and in vivo experiments [167,230]. The same overexpression of miR-148a with glabridin treatment was observed to be correlated with suppression of Wnt/β-catenin signaling pathway, which in turn attenuates angiogenesis and vascular endothelial grow factor (VEGF) secretion [166].

### 5.3. Coumestans

Coumestrol is considered a chemoprevention agent in estrogen-responsive carcinomas [70], and a potential therapeutic agent in other pathologies such as neurological and autoimmune disorders [56]. Antitumorigenic activities of coumestrol include anti-proliferative effect, inhibition of apoptosis and oncogenesis which had been demonstrated in vitro breast, cervix, lung and prostate cancer models [297,298]. The mechanism beyond its antitumor activities is not entirely elucidated. Recently, coumestrol had been discovered to act as novel inhibitor of protein casein kinase 2 (CK2), which is a highly expressed in several human cancers, including breast, ovarian and cervical neoplasia [161]. CK2 is a serine/threonine protein kinase that phosphorylates PTEN resulting in increased PI3K/AKT signaling in ovarian cancer. By blocking the CK2 activity, coumestrol can potentially target several keys signaling pathways such as the PI3K/AKT and ERK1/2 MAPK [161]. Interestingly, CK2 phosphorylates the DNA methyltransferase 3A, and thereby coumestrol can downregulate the activity of DNMT3A. Genome-wide DNA methylation analysis showed that CK2 modulates CpG methylation of several repeats, and that CK2-mediated phosphorylation is required for localization of DNMT3A to heterochromatin [299]. Recently, a study reported that coumestrol had inhibitory effect on Haspin kinase activity and downregulated phosphorylation level of histone H3Tr3, in colon cancer cells [162]. Haspin is a protein kinase that phosphorylates histone H3 at Thr3 residue and this epigenetic marker is necessary for cellular mitosis progression [300]. Depletion of Haispin kinase and as a consequence lack of phosphorylation at H3Thr3 induces chromosome alignment defects and failure of mitosis [300,301]. Thereby, coumestrol can indirectly affect the epigenome by reshaping the CpG methylome and modulating the H3 phosphorylation pattern through its inhibitory activities against protein kinases.

Interestingly, coumestrol had been able to induce mitochondrial biogenesis, by activating the SIRT1 in cultured myocytes. A high increase of mitochondrial contents along with increased expression of key proteins in the mitochondrial electron transfer chain and an elevation of ATP concentration was observed. By inhibiting the SIRT1 expression the effects induced by coumestrol on mitochondrial biogenesis were abolished [239]. Recent data confirm that the SIRT1 could promote mitochondrial biogenesis in conditions of energy deficiency associated with disease and injury [302].

Wedelolactone, a component of coumestans family has a high affinity for EED, and in vitro blocks the EZH2-EED interaction and inhibits PRC2-complex that is responsible for tri-methylation of H3 histone at lysine 27 position (H3K27me3) [241]. This histone epigenetic mark and PRC2-complex are playing an important role on carcinogenesis, and also in tissue differentiation in normal development [303]. Therefore, some PRC2-dependent cancer cells undergone growth arrest upon treatment with wedelolactone and several tumor suppressors, downstream targets of PRC2 were activated [241]. Moreover, in vitro experiments using wedelolactone at physiological concentration, have demonstrated an inhibitory effect of wedelolactone on histone methytransferase activity of EZH2 and a decrease of EZH2 protein expression [240]. These activities were correlated with a strong anti-proliferative effect on non-Hodgkin’s lymphoma cells [240]. The anticancer capacity of wedelolactone has been observed in vitro and in vivo in several cancer models and could be explained by its ability to bind and inhibit the PRC2 complex.

### 5.4. Lignans

Lignans are phytoestrogens with a significant potential on human health, and with diverse biological activities. Still their potential capability to modulate the epigenome and epigenetic mechanisms just started to be investigated. [171]. The flaxseed lignan SDG and its gut metabolite, ENL have been studied in murine adipocytes exposed to reactive oxygen species and an interconnection between antioxidant potential and their capacity to modulate the epigenome was observed. The expression of epigenetic modifier genes DNMTs, HDAC1/2 and MBD2 was downregulated significantly in control adipocytes and hydroxyl-radical-treated adipocytes in comparison with superoxide-radical-treated cells [248]. Hydroxyl radicals are highly reactive and cause DNA modifications which might result in the oxidative damage of DNA and epigenetic alterations that affect chromatin organization [304]. The downregulation of several epigenetic modifier proteins would prevent gene silencing and the occurrence of epigenetic alterations produced by hydroxyl radicals. Moreover, the strong anti-proliferative effect of ENL on mid-and late-stage models of prostate cancers has been reported [252]. These effects were associated with significantly reduced expression of the miR-106b cluster (miR-106b, miR-93 and miR-25) and oncogene MCM7, and with the upregulation of the PTEN tumor-suppressor gene [252]. The highly conservative miR-106b cluster is located within an intron of MCM7 gene and is overexpressed in several human malignancies, including breast and prostate cancers [305]. Emerging evidence have been demonstrated that miR-106b cluster promotes tumorigenesis by regulating multiple cellular processes associated with cancer development and progression [305]. Moreover, a flaxseed diet which exerts antioxidant and anti-inflammatory capacity in preclinical disease models can significantly modulated expression of multiple miRNAs, including miR-142-3p, miR-150 and miR-34a with complementary role in tumor suppression and cancer radiosensitization [250].

Recently, a comprehensive study compared the effect of flaxseed as a whole food with its isolated components, flaxseed oil and SDG, on healthy female mice during mammary gland development. The study has identified a diet-specific miRNA signatures and a diet-dependent deregulated expression of miRNAs associated with breast cancer [249]. As other studies have showed that a SDG-based diet during pregnancy and lactation helped in reducing the susceptibility of small animals to mammary carcinogenesis and reduced tumorigenesis [249].

Other dietary lignan, sesamin, has exerted its anti-inflammatory effect by suppressing macrophage-derived chemokine expression in human immune cells [251]. The intracellular mechanism has revealed that sesamin inhibited MAPK-p38 and NFkB-p65 pathways through epigenetic modulation of H3 and H4 acetylation in the macrophage-derived chemokine promoter area. The hypo-acetylated pattern at H3 and H4 induced by sesamin could be a result of decreasing recruitment of CBP histone acetyltransferase protein associated to NFkB-p65 subunit [251]. Interestingly, the suppressive effect of sesamin on both MAPK-p38/NFkB-p65 pathways was also reported in several cancer cells and animal models [306], indicating that sesamin might be a potent inhibitor of these two protein complexes.

Arctigenin induces apoptosis of ER-negative breast cancer in vitro and in vivo through a ROS mediated MAPK-p38 pathway and by epigenetic regulating the Bcl-2 expression through increasing H3K9 trimethylation [169]. The H3K9 trimethylation at promoter region is often an epigenetic mark for silencing transcription and maintaining the chromatin in condensed state [307].

Several studies have reported the antioxidant and anti-inflammatory activities of arctigenin in different pathologies. In cerebral and myocardial ischemia, the same mode of action of arctigenin has been reported, through activation of SIRT1 signaling pathway [242,244]. From in vivo studies of myocardial ischemia, arctigenin seemed to activate AMPK/SIRT1 signaling pathway, and consequently an upregulation of I-κB and inhibition of NF-κB have been observed. Thereby, it has been able to reduce oxidative stress, inflammation and apoptosis in cardiomyocytes, through SIRT1 signaling pathways [244]. Moreover, in vivo and in vitro experiments have demonstrated that arctigenin treatment effectively inhibited cerebral ischemia, by inducing NLRP3 inflammasome activation and IL-1β, IL-18 secretion and by activation SIRT1 [242]. Moreover, a recent study has demonstrated that it can modulate the miRNAs expression against neuroblastoma cells by reducing expression of inflammation genes TNF-α and IL-6, and increasing expression of anti-inflammatory protein IL-10 [243]. The miR-16 and miR-199a have been positively regulated by arctigenin to reduce expression of direct upstream activators, IKKα and IKKβ of NF-κB signaling pathway, thereby inhibiting their activation as well as pro-inflammatory cytokines (IL-6 and TNF-α) activities [243]. Notably, bioinformatic analysis suggested that one of the miR-199a potential gene target is SIRT1 [144].

In vivo experiments have showed that arctigenin at physiological concentration could modulate multiple-responsive signaling molecules involved in proliferation, apoptosis, angiogenesis and invasion of prostate cancer through miRNAs expression [245]. Nevertheless, the arctigenin glucoside derivative, arctiin is also capable to modulate miRNAs expression in order to promote UVB protective effect on human dermal fibroblasts and keratinocytes [246,247] Identifying miRNAs that have their expression modulated by these phytoestrogens may lead to our understanding of the regulatory mechanisms mediated by their target mRNAs and will facilitate the efforts to maximize the therapeutic benefits of phytochemicals. The molecular mechanism that connects these regulated miRNAs and the anticancer function of arctigenin remains to be elucidated.

### 5.5. Stilbenes

The effect of stilbenes on the epigenome has been intensely studied in last decade. Specifically, resveratrol has been in the center of scientific studies due to its therapeutic potential in many diseases, including cancer, diabetes, cardiovascular and neurodegenerative diseases and metabolic disorders [267]. Resveratrol can act at every level of epigenetic mechanisms: DNA methylation, histone acetylation and methylation, chromatin remodeling and non-coding RNA expression, as Table 2 shows [176,185].

Genome-wide technologies have proved that stilbenoids can remodel patterns of DNA methylation in human cancer and normal cells, leading to CpG-loci specific hypermethylation or hypomethylation [259,262]. Notably, the results on normal mammary cells studies showed that genes with methylated sites or regions were enriched with pathways associated with chronic disease prevention mechanisms [259]. DNA methylation changes in normal cells and blood of healthy animals have occurred upon prolonged exposure to stilbenoid compounds at physiological concentrations [259]. Thus, it can be assumed that these subtle epigenetic modulations in normal cells are necessary for maintaining a healthy phenotype. In vivo experiments with prolong administration of resveratrol (21 weeks) to animal models bearing breast tumors showed a difference in DNMT3B expression levels between normal and tumor mammary tissues [279]. This different pattern of DNMT3B expression highlighted once again that resveratrol and other stilbenes could differentially modulate normal versus malignant tissue gene expression. Moreover, in human studies, the glucuronide metabolite of resveratrol had a dose-related effect on DNA methylation of RASSF-1α and prostaglandin expression [308].

In contrast to genistein, resveratrol can act on all three enzymes that control the epigenetic process of DNA methylation. It cans downregulate DNMT1 expression, by inhibiting its activity in chronic models in vitro and in vivo. Studies revealed that resveratrol can demethylate and elevate expression of PTEN, which subsequently blocks the AP-1 presence at the DNMT1 gene promoter [309]. By downregulating DNMT1 and increasing PTEN expression, a cascade of events starts and as a result Ras/Raf/MAPK/AP-1 signaling pathway is inhibited and ER is activated [310]. In breast cancers, resveratrol inhibits by hypermethylation the expression of MAML2, which acts as a transcriptional coactivator for NOTCH proteins [286]. This effect was correlated with a higher presence of DNMT3B protein and a reduced binding of OCT1 transcription factor at enhanced region of MAML2 gene [286]. Thus, the presence of DNMT3B hinders the binding of transcriptional factors to oncogenic genes enhancing the methylation and inducing onco-genes silencing. Epigenetic regulation of the JAK1/STAT3 pathway is common in many tumor cells. The phosphorylation and acetylation of STAT3 are crucial events for STAT3-mediated upregulation of oncogenic genes and interplay between DNMT1 with role in maintaining the methylation at promoter genes, and DNMT3B the novo methyltransferase has a key role. It has been suggested that the inhibition of carcinogenesis can be mediated through STAT3 acetylation by p300 and DNMT3B-dependent silencing of a protein tyrosine phosphatase promoter, which is succeed by a long-term preservation mediated by a DNMT1-dependent loss of JAK1 kinase activity [311]. Resveratrol had shown inhibitory activity on STAT3 acetylation, and it consequently reactivated several tumor suppressors’ genes, such as ESR1 in breast cancer and melanoma [255]. By inducing deacetylation of oncogenic transcription factors, resveratrol can concurrently promote demethylation of CpG island of tumor suppressors to inhibit cancer development and progression.

Furthermore, combination of resveratrol and pterostilbene treatment on triple-negative breast cancer cells resulted in enzymatic activities inhibition of DNMTs, HDACs, downregulation of DNMTs and SIRT1 expression and global DNA hypomethylation [261,262]. In addition, the modulation of several epigenetic alterations was accompanied by a decrease in DNA damage response enzymes, resulting in cell-cycle arrest, along with apoptosis induction [261]. In general, stilbenes can induce intense hypermethylation at promoter genes with oncogenic and pro-metastatic functions in several signaling pathways, such as NOTCH, WNT, MAPK and JAK/STAT, as is common in several types of cancers [175,269,286].

Resveratrol epigenetically regulates Nrf2 expression, exerting its chemoprotective properties on estrogen-induced breast cancer by demethylation of Nrf2 promoter and thus reactivates the downstream antioxidant genes [272]. On the other hand, in vitro and in vivo experiments have described resveratrol as an activator of SIRT1 deacetylase activity, and many of its biological activities are closely related to this protein. Whilst SIRT1 expression is lost or its activity is inhibited, resveratrol’s biological activity is partially abolished [179]. As versatile bioactive molecules, resveratrol has multiple molecular targets, and it is reasonable to assume that it can inhibit classical HDACs and also to sustain the activation of SIRT1. Interestingly, resveratrol is also capable to induce a nitric oxide–dependent crosstalk among SIRT1 and HDAC2, by activating SIRT1 and stimulating NO synthesis and also reducing binding capacity and activity of HDAC2 in skin repair model experiments [253]. Moreover, instead of directly activating SIRT1, resveratrol may modulate SIRT1 activity via another target protein, AMPK with main role in cellular energy homeostasis [312], or enhances the SIRT1 activity by facilitating laminin A binding to SIRT1, and serving as an allosteric effector of SIRT1 [313]. In vivo experiments have shown that resveratrol is able to stimulate mitochondrial biogenesis and functions, by activating SIRT1 which in turn deacetylates LKB1 and activates AMPK [265]. The concentration of resveratrol is a critical parameter and its capacity to modulate mitochondrial NAD+/NADH ratio along with SIRT1 activity can provide health benefits by activating SIRT1 downstream pathways in metabolic syndrome and age-related diseases [265,314]. As resveratrol and pterostibenes, the piceatannol and its *O*-methyl metabolite isorhapontigenin have demonstrated a strong capacity to upregulate SIRT1 mRNA and SIRT1 protein in monocytes cells [282], and have shown a strong effect on miRNAs expression in melanoma and colon cancer [283,284]. Given free piceatannol’s stability in plasma and the biological activity of its methylated metabolite, they may have a more impact on human health than less stable and poor bioavailable resveratrol.

All known stilbenes exert their antioxidative and anti-inflammatory activities via regulating several signaling pathways involved in disease prevention mechanism. Recently, it has been demonstrated that they can modulate inflammation through modulating miRNAs expression. Resveratrol induced the upregulation of miR-663, a miRNA associated with immune response modulation, which in turn decreased AP-1 activity and impaired the increase of the pro-inflammatory miR-155, with oncogenic activity in human monocytes cells [268,280]. Several studies have demonstrated that resveratrol can modulate the expression of miR-663 and its target the transforming growth factor beta 1 (TGF1) transcript, in inflammation and inflammation-related cancers [277,315]. Moreover, miR-663 is considered to be a mediator of resveratrol anti-inflammatory activity [316]. Moreover, the stilbenes anti-proliferative and pro-apoptotic effects on cancer cells are closely related to their strong capacity to modulate tumor suppressors’ miRNAs that control pathways of cell death and cell cycle [317]. For example, in breast cancer cell lines resveratrol modulates miR-122-5p, miR-125b-5p, miR-200c-3p, miR-409-3p and miR-542-3p expression, with impact on Bcl-2, X-linked of apoptosis protein (XIAP) and CDKs, that are proteins regulating the cell cycle [317]. Both resveratrol and pterostilbene are able to downregulate the PTEN-targeting members of the oncogenic miR-17 family of miRNAs, which are overexpressed in prostate cancer [275]. Moreover, the pterostilbene treatment had led to reduction of prostate tumor growth, and also had decreased the levels of circulating oncogenic miRs-17 and -106a, showing that stilbene’s chemopreventive effect could be related to their epigenetic modulator capacity [275].

In conclusion many biological activities of phytoestrogens can rely on their epigenetic modulator capacity, yet many aspects regarding their direct or indirect interaction with the epigenome and the molecular mechanism underlying their action should be investigated.

## 6. Future Perspective and Conclusions

Increasing scientific evidence is suggesting that dietary patterns have an impact on human health and life expectancy by their potential role in preventing of chronic diseases [2,17,309]. A diet rich in raw fruits and vegetables supplies the human body with biological active compounds including phytoestrogens that can act as agonist or antagonist estrogenic molecules, antioxidants and anti-inflammatory nutrients. Thus, they are being able to activate protective mechanisms. Many phytoestrogens are consumed at every meal, several times per day, over long terms, thus increasing the possibility of accumulating or repeatedly producing beneficial or adverse effects on human health. However, the optimal concentration, mode of administration and frequency of use of dietary phytoestrogens to produce beneficial effects are far from being completely understood.

In general, their low absorption and bioavailability has been cited as a reason against their possible beneficial effects on health. However, the low bioavailability of phytoestrogens is consistent with the concept that these molecules may produce beneficial hormetic effects at low doses. The term “hormesis” describes the phenomenon where a specific compound is able to induce biologically opposite effects at different doses; it most commonly has a stimulatory or beneficial effect at low doses and an inhibitory or toxic effect at high doses [318]. Notably, in vitro studies have revealed that phytoestrogens are capable to induce biphasic dose–response on several types of cell lines, especially tumorigenic [188,202,203]. The fact that, at low concentrations, most of the phytoestrogens stimulated the proliferation rate of cancer cell lines can have an important impact on carcinogenesis. However, at high concentrations of phytoestrogens, the apoptotic events were observed [189,193,270]. Importantly, the working concentrations of phytoestrogens with a stimulatory effect on proliferation of cancer cells are, in many cases, much higher than the physiological ones and could not be relevant for the whole organism or for various organs targeted by bioavailable phytoestrogens. Even so, there are examples of phytoestrogens that have anti-proliferative and anticancer activities at low concentrations. Genistein exerts its preventative effects by epigenetically modulating BRCA1 gene’s CpG methylation and downregulating its expression in ERα positive breast cancer cells with activated AhR [190]. Recently, the treatment of breast cancer cell lines with 6-PN at 1 μM concentration has been able to activate AhR in order to attenuate epigenetic inhibition of CYP1A1 through degradation of ERα [228]. Moreover, the daidzein metabolite S-equol, at a low concentration and for long-term exposure (three weeks), has demonstrated a significant anti-proliferative effect on triple-negative-breast-cancer cell lines, along with epigenetic modulator capacity of DNA methylation at BRCAs promoters [192]. Remarkably, many dietary phytoestrogens and their metabolites at human plasma concentrations are able to reduce oxidative stress or inflammatory response in vitro and in vivo models [204,224,231,232,242,244].

As this review shows, most phytoestrogens have multitudinous molecular targets, including epigenome marks, which can influence the transcriptional status of several genes. The direct interactions of phytoestrogens with proteins that are controlling the epigenetic mechanisms have been demonstrated, such as the inhibitory effect as direct binding to DNMTs or HDACs [189,229,260]. However, intense investigations are still needed to decipher the molecular mechanisms beyond their epigenetic modulator capacity, especially their impact on non-coding RNAs expression. Given that there are many examples of dietary phytoestrogens that are biologically active while provided at a low dose for a sustained period of time [173,259,260,278,281], rather than at higher doses for a shorter period, it is possible that the wide range of beneficial properties may rely on their capacity to simultaneously reset or activate the expression of multiple miRNAs or lncRNAs. In addition, the synergistic or additive effects of different phytoestrogens and other biologically active phytochemicals present in the human diet may be of significant importance and should be explored in future studies. The interconnection between the phytoestrogens’ action at epigenetic levels and their capacity to activate multiple pathways related with inflammatory and antioxidant response should be an approach to find out the health benefit that they can induce.

Another important aspect of phytoestrogens impact on humans is regarding how inter-individual genetic, epigenetic and gut microbiota variability might influence their mode of action. At this moment, the dietary phytoestrogens have proved to promote intestinal homeostasis by improving the gut barrier’s integrity and short-chain fatty acids’ production and by modulating the composition of the gut microbiota [13,16,17]. As the gut microbiota of individuals are highly variable and difficult to standardize, establishing a metabolomics profile of urine and fecal samples with regards to phytoestrogens consumption will help to understand the variation between the microbial composition and therapeutic effects of phytoestrogens. Future studies with particular attention to the action of phytoestrogen metabolites are required for better understanding of mechanisms behind their beneficial or adverse effects. Measuring phytoestrogens’ exposure by means of dietary assessments or by their intake in clinical studies does not reflect the actual presence of phytoestrogens in the organism, since it does not comprise the biotransformation of them by the gut microbiota and their absorption. In those studies, it is not possible to unveil the effects of compounds produced by microbiota, such as S-equol, *O*-DMA, enterolignans and lunularin. Within this scenario, the measurement of bioactive phytoestrogens and their metabolites as biomarkers in blood or urine samples seems a better approach in order to have an objective and more accurate estimation of phytoestrogens exposure. Hence, the clinical study design should include more randomized controlled trials, different doses of phytoestrogens tested and even genotyping and microbiota profile of enrolled individuals. 

New developments in metabolomics field are now capable to identify biomarkers of dietary patterns and combined with modern statistical methods; it might provide more insights into the relationships between diet and disease prevention. Future studies based on novel approaches, such as genome-wide association studies, metabolomics and nutritional epigenetics, will provide more scientific clinical evidence of dietary phytoestrogens’ impact on human health.

## Figures and Tables

**Table 1:** Dietary source of phytoestrogens (mg/100 g or mg/100 mL).

Source	Isoflavones	Coumestans	Prenylflavonoids	Lignans	Stilbenes	Total	References
I. Soy and processed soy products							
whole soybean	5.47–159.61	0.0015–0.225	N/D	0.154–0.270	N/D	5.625–160.11	[4,65]
soybean sprout	0.674–14.05	0.1–0.34	N/D	0.12–0.15	N/D	0.894–14.54	[4,65]
roasted soybean	148.5–246.95	0.02–0.03	N/D	0.09	N/D	148.61–247.07	[4,65]
tofu (incl. fermented)	22.7–48.51	0.0007–0.12	N/D	0.09–0.16	N/D	22.79–48.79	[4,63,134]
edamame (fresh soybeans)	44.99–48.95	N/D	N/D	0.3	N/D	45.29–49.25	[4,65,134]
tempe (fermented soybeans)	60.61–147.74	0.0006	N/D	0.01–0.02	N/D	60.62–147.76	[4,65,134]
miso paste (fermented soybeans)	41.15–63.09	0.00024–0.04	N/D	0.02–0.03	N/D	41.17–63.16	[4,134]
II. Seeds, bread and cereal							
flax seeds	0.07–0.321	0.03–0.047	N/D	0.013–301.13	N/D	0.113–301.497	[4,134,135]
sesame seeds	N/D	0.0004	N/D	39.348–74.95	N/D	39.35–74.95	[134]
granola	0.02–93.9	N/ D	N/D	0.21–0.764	N/D	0.23–94.664	[134,136]
sunflower seeds	N/D	0.002–0.01	N/D	0.891–2.10	N/D	0.893–2.11	[63,134]
pumpkin seeds	0.017–0.018	N/D	N/D	0.52	N/D	0.537–0.538	[135]
flax and/or soy-containing bread	0.297–14.67	0.01–0.09	N/D	0.01–1.379	N/D	0.317–16.139	[134,136]
III. Nuts							
almond	0.01–0.044	0.02	N/D	0.112–0.92	N/D	0.142–0.984	[63,65]
Brazil nuts	0.105–0.109	0.01–0.02	N/D	0.77–0.782	N/D	0.885–0.911	[63,135]
cashew	0.01–0.023	0.0004	N/D	0.17–56.33	N/D	0.180–56.353	[4,134,135]
peanut	0.02–0.57	0.0001–0.002	N/D	0.026–6.803	0.071–0.178	0.117–7.553	[135]
pistachios	0.033–3.63	0.007–0.01	N/D	0.029–0.19	0.009–0.167	0.078–3.997	[135]
walnut	0.03–7.9	0.0006	N/D	0.086–0.13	N/D	0.117–8.031	[63,135]
IV. Vegetables							
alfalfa sprout	0.39–5.507	0.0025–0.105	0.045	0.02–0.0448	N/D	0.458–5.702	[4]
broccoli	0.044–0.134	N/D	N/D	0.787–130.01	N/D	0.831–130.14	[4,134]
Brussel sprout	N/D	0.04	N/D	50.36–72.36	N/D	50.40–72.40	[136,137]
cabbage	0.0031	N/D	N/D	0.03–78.31	N/D	0.033–78.31	[4,65]
carrot	0.0052–0.0066	0.001–0.0014	N/D	0.006–7.66	N/D	0.012–7.67	[65,137]
cauliflower	2.3–6.7	0.8–1.8	N/D	25.2–145.1	N/D	28.30–153.60	[65]
green bean	0.04–0.718	N/D	N/D	0.065–23.07	N/D	0.105–23.79	[63,65]
sauerkraut	N/D	N/D	N/D	18.3–31.6	N/D	18.3–31.6	[65]
V. Fruits							
apple	0.0009–0.067	0.0011	N/D	0.0012–0.1	0.0395–0.0956	0.0427–0.2637	[134]
apricot	0.0070	N/D	N/D	0.22–42.97	N/D	0.227–42.977	[4,65]
dried apricot	0.0390	0.0042	N/D	0.4	N/D	0.443	[4]
coconut	0.02–44.107	N/D	N/D	0.023–0.032	N/D	0.043–44.139	[135]
grape	0.0350	N/D	N/D	0.029–5.4	0.345	0.409–5.780	[4]
orange	0.002–0.0024	0.013–0.053	N/D	0.078–2.71	N/D	0.093–2.765	[65]
pear	0.0007–0.027	N/D	N/D	0.038–18.96	0.0276–0.0502	0.066–19.037	[4,65]
plum	0.007–0.015	N/D	N/D	0.005–0.589	0.019–0.048	0.031–0.652	[65,134]
dried plums	0.0042	0.0018	N/D	0.1775	N/D	0.184	[4]
VI. Beverages							
beer	0.0015–0.13	N/D	0.044–5.189	0.01–0.63	0.01–1.479	0.066–7.428	[65,134]
cocoa	N/D	N/D	N/D	0.03–0.3	0.14–0.23	0.170–0.530	[63]
coffee	0.04–0.051	0.03	N/D	0.01–6.41	N/D	0.08–6.491	[134,135]
tea leaves/infusion	0.007–0.05	0.03	N/D	0.022–3.12	N/D	0.059–3.2	[65]
red wine	0.001–0.02	0.0001	0.85	0.134–1.2	0.78–4.35	1.765–6.420	[65,134]
white wine	0.001–0.024	0.0001	0.23	0.008–0.4	1.05	1.289–1.704	[65,134]

N/D = compounds not detected; single values should be considered as the maximum detected quantity.

**Table 2:** Estrogenic effects of dietary phytoestrogens.

Phytoestrogens		Relative Binding Affinity to ERs	Estrogenic Effects	References
Isoflavones	Daidzein	-Preferential affinity to ERβ in comparison with ERα (RBA 0.5 v 0.1); -Lowest binding affinity of all isoflavones.	-Inhibits ovarian cancer cells’ proliferation, migration, and induces cell-cycle arrest as selective ERβ agonist; -Chemoprevention in endometrial cancer.	[6,7]
	Genistein	-Higher affinity to ERβ in comparison with ERα (RBA 31–87 v 1–4).	-Regulated expression of target endogenous genes (CYP17, PR, ER-α, and ER-β). -Modulates ER levels in the liver, testis and lung; -Inhibits aromatase expression in breast cancer tissue; -Enhances osteoblastic differentiation and maturation by activation of ER; -Effects on reproductive and nonreproductive organs of mice models; -Preventive effect on breast and prostate cancers; -Decreases ovarian cancer risk;	[6,7,70]
	S-equol	-Preferentially binding to ERβ (RBA 10× higher for ERβ than ERα); -Higher binding affinity than its precursor daidzein.	-Binds dihydrotestosterone and inhibits in vivo prostate cancer growth; -Potent agent for menopause-related symptom relief.	[19,160]
Comestans	Coumestrol	-High binding capacity, similar to estradiol (RBA for ERβ: 77–140 and for ERα: 12–20).	-Inhibits aromatase and 3α-hydroxysteroid dehydrogenase activities; -Anticancer for ovarian, breast, lung, cervical and prostate, cancers through ER signaling pathways; -decreased endometrial cancer risk;	[161,162]
	Wedelolactone	-Acts as an agonist to both ERs.	-Activates expression of ER-regulated genes in ER-positive breast cancer cells; -Stimulates transactivation by AP-1 on ER signaling pathway.	[163]
Prenylflavonoids	6-PN	-Very weak binding capacity to ERα and ERβ (less than 1/100 of 8PN RBA to ERβ; -Weak aromatase inhibitor;	-Alters ERα-AhR crosstalk under estrogenic conditions; -Preferentially induce non-genotoxic estrogen metabolism	[164,165]
	8-PN	-Binds tighter to ERα compared to ERβ; -Acting as a pure estrogen with activity similar to estrone; -Potent aromatase inhibitor;	-Reduces the raised skin temperatures in a menopausal rat model; -Reduces hot flashes in postmenopausal women; -Increases uterine weight and height of luminal epithelial cells; -Weak osteoprotective effects;	[164,165]
	Glabridin	-Binds to ERβ with the same affinity as genistein; -Weak binding affinity to ERα.	-Induced dose-dependent increase in estrogenic activity and cell proliferation in Ishikawa cells; -Exerts estrogenic activity via the ER-α-SRC-1-co-activator complex; -Acts through the ER to induce beneficial effects of estrogen in bone and cardiovascular tissues.	[166,167]
Lignans	Arctigenin	-Moderate binding capacity for ERβ; -Weak binding affinity to ERα.	-Inhibits the activation of mTORC1 pathway by targeting ERβ; -Promotes ERβ activation in T lymphocytes	[168,169]
	Enterolactone	-Weak binding affinity to ERα and ERβ;	-Induces ERα/β transcriptional activation; -Activates estrogen-responsive genes, through direct binding to the ligand-binding domains of ERα; -Increases the expression of endogenous estrogen-responsive genes in mouse uterus. -Reduces the risk of hormone-dependent breast, uterus and prostate cancers.	[170,171]
Stilbenes	Resveratrol	-Binds with similar affinity to ER-α and ER-β; -Weak estrogenic binding ability in comparison with estradiol (~7000 times less powerful).	-Inhibits endometrial aromatase and COX-2 expression; -Suppresses vascular smooth muscle proliferation and promotes re-endothelialization after aorta injury; -Mediates its neuroprotective effects via ER activation; -Interferes with intestinal and hepatic metabolism of estrogens	[121,123]
	Pterostilbenes	-Binds with approximatively similar affinity to ER-α and ER-β.	-Inhibits colon cancer tumors growth in mice models through an ER-β-mediated mechanism; -Induces apoptosis in breast cancer cells and necrosis in xenograft tumors by targeting ER-α36; -Enhances the sex hormone secretion, further improving follicle development.	[172,173]
RBA, Relative Binding Affinity.				

**Table 3:** Dietary phytoestrogens as epigenetic modulators—in vitro and in vivo studies.

Dietary Phytoestrogen	Concentration	Cell Line/In Vivo Models	Epigenetic Changes	Targeted Gene	Biological Activity	References
I. Isoflavones						
Genistein	In vitro 0.5–1 μM	MCF-7	↓DNMT1, ↓CpG methylation at BRCA1, ESR1 promoter	↑BRCA1, p53, CYP1A1, ↓cyclin D1	-Anti-proiferative and chemopreventive effect in breast cancer cells with activated AhR.	[190]
	3.125 μM	MDA-MB-468,	↓CpG methylation at GSTP1 promoter	↑GSTP1	-Preventive effect, activates phaseI enzyme in TNBC cells.	[211]
	5 μM	MDA-MB-435, Hs578T	↓miR-155	↑FOXO3, PTEN, casein kinase, p27, ↓β-catenin	-Inhibits cells viability and induces apoptosis in TNBC cells.	[197]
	18.5 μM	MCF-7 MDA-MB-231	↓Global DNA methylation, ↓CpG methylation BRCA1, BRCA2, MeCP2 promoters; ↓H3K9me3, H3K4me3, H3K27me3; ↑H4K8ac, H3K4ac at promoters of EZH2,BRCA1, ERα, ERβ, SRC3, p300	↑BRCA1, ↑BRCA2; ↓EZH2, ↑p300, ↑SRC3	-Inhibition of breast cancer cells’ proliferation.	[191,195]
	10–20 μM	UACC-3199, KYSE 510, SiHa; DU145, LNCaP, PC-3, ARCaP-E, ARCaP-M	↓DNMT1, ↓CpG methylation of BRCA1, ESR1 promoters; ↓CpG methylation at RARβ2, p16, MGMT, ↓DNMTs; ↓DNA methylation at RARβ 2; ↑H3K9ac at promoters of APC, SOX7, SFRP1, SFRP2, DKK, WIF1, ↑HAT1	↑BRCA1, ER α, p53 CYP1A1, ↓cyclin D1 ↑RARβ2, p16, MGMT ↑RARβ2; ↑SOX7, SFRP1, BRCA1, BARD1, RAD23B, XRCC2, ↓BIRC7, SLUG, HES1, TGFBIII	-Anti-proiferative and chemopreventive effect in breast cancer cells with activated AhR; -Inhibition of esophageal squamous cell carcinoma growth; -Induces apoptosis in cervix squamous cells carcinoma; -Reduces proliferation and induces apoptosis in prostate cancer cells	[190,193,212,213]
	25 μM	MDA-MB-157, MDA-MB-231; A-498; 786-O; Caki-2; PC3, DU145, RWPE-1; 786-O, ACHN; PC-3, DU145	↑Ac-H3, ↓DNMT1, HDAC1; ↓miR-1260b; ↓CpG methylation at SFRP1 ↓H3K9me2, H2K9me3, H3K27me3 at SFRP1, Smad4 genes; ↓lnc HOTAIR, ↓EZH2, ↑miR-574-3p, ↑mir-34a	↑Erα; ↑SFRP1, Dkk2, Smad4; ↓ARID1A, EED SMARCB1, SNAIL, ↑ZO-1; ↓RAC1, ↓EGFR, ↓EP300 ↓MMP9, VEGF	-Inhibitits proliferation, invasion of TNBC, renal carcinoma and prostate cancer cells; -Enhances tamoxifen induced anticancer effect in TNBC cells; -Promotes apoptosis in renal carcinoma and prostate cancer cells; -Inhibits cell proliferation, migration and invasion in vitro and in vivo in prostate cancer;	[196,199,200,210,214,215]
	5–40 μM	SH, SHR	↑Global H3ac, ↑H3K4me3, ↓H3K27me3, H3K9me3 at p16, p21 promoters, ↑HMTs activities	↑p21, p16, ↓BMI1, c-MYC	-Inhibits growth of breast cancer cells, but no effect on normal cells; -Preventive effect on breast tumorigenesis in vivo.	[188]
	20–40 μM	U266	↑miR-29b	↓NF-κB	-Inhibits proliferation and induces apoptosis in multiple myeloma.	[216]
	40 µM	DU-145, PC-3 LNCaP	↓CpG methylation at BRCA1, GSTP1, EPHB2, RASSF1A promoters; altered methylation pattern of MAD1L1, TRAF7, KDM4B, hTERT genes; ↑miR-200c	↑ BRCA1, GSTP1, ↑EPHB2; Potential target genes SOX2, ZEB1	-Inhibits prostate cancer cells’ proliferation, clonogenic potential and induces apoptosis.	[187,217,218]
	50 µM	HeLa	↓DNA methylation at TP53, PTEN, CDH1, DAPK1, FHIT, RUNX3, SOCS1 promoters, ↓DNMTs, HDACs, HMTs	↑TP53, PTEN, CDH1, DAPK1, FHIT, RUNX3, SOCS1	-Anti-proliferative effect on cervical tumor cells.	[194]
	60–100 μM	MCF-7, MDA-MB-231	↓Global DNA methylation; ↓DNAmethylation at TSG promoters; ↓DNMT1	↑ATM, ↑APC,↑PTEN	-Reduced cellular viability and anti-proliferative effect on breast cancer cells;	[189]
	In vivo 2 mg/day 50 mg /kg 250 mg/kg 270 mg/kg	Neuroblastoma xenografts, Eker rats, A^vy^ female mice, tumor xenograft mice, 123/SvJ:C57BL/6J mice	↓DNTM3B, ↓CHD5 promoter methylation; ↓EZH2, ↓H3K27me3; ↑CpG methylation at A^vy^ IAP gene; ↓DNMT1, HDAC1; ↓HDACs activity; ↑DNA methylation in repetitive elements; ↓HDAC6	↑CHD5; ↑PI3K/AKT pathways in uterus; ↓ectopic Agouti expression; ↑ERα, ↑PCNA in breast tissues; ↓p21, cyclin D1, PCNA, IGF2 expression in adult mice	-Decrease of tumor size and frequency. -Increases hypersensivity of ER-responsive genes in neonatal uteri and adult myometrium; -Reduces obesity offsprings, phenotypes changes; -Re-sensitizing ERα-negative breast cancer to therapy; -Prenatal exposure leads to long-term epigenetics changes.	[196,219,220,221,222]
	Soy based diet	Cynomolgus monkeys	↓DNA methylation at promoter of HOXA5, HOXA11, HOXB1, ABCG5	↑HOXA5, HOXA11, HOXB1, ABCG5	-Decrease in fasting insulin and HOMA index values.	[223]
Daidzein	20–50 μM	KYSE 510	↓DNA methylation at RARβ2 promoter; ↓DNMTs activity	↑RARβ2	-Dose-dependent inhibition of cells growth.	[193]
	78.5 μM	MCF-7, MDA-MB 231	↓Global DNA methylation, ↓CpG methylation at BRCA1, BRCA2 promoters, ↓MeCP2; ↓H3K9, H3K27, H3K4 (me3) ↑H4K8ac, H3K4ac at promoters of EZH2, BRCA1, ERα, ERβ, SRC3, p300	↑BRCA1, BRCA2; ↓EZH2, ↑p300, SRC3	-Inhibition of ER(-) and ER(+) breast cancer cells’ proliferation.	[191,195]
	110 µM	DU-145, PC-3 LNCaP	↓CpG methylation at BRCA1, GSTP1, EPHB2 promoters; altered methylation pattern of MAD1L1, TRAF7, KDM4B, hTERT	↑BRCA1, GSTP1, EPHB2	-Inhibits prostate cancer cells’ proliferation and induces apoptosis.	[187,218]
Biochanin A	2–6 μM	T47-D, MCF-7	↑miR-375	↑ERα, ↑Bcl -2	-Promotes proliferation of breast cancer cells.	[203]
	2.95 μM	MGC-803	↓LSD1, ↑H3K4me1/2, H3K9 me1/2	↓MAO-A/B, Bcl-2, ↑Bax	-Suppresses colony formation and migration and induces apoptosis in gastric cancer cells.	[201]
	20–50 μM	KYSE 510	↓CpG methylation at RARβ promoter, ↓DNMTs activity	↑RARβ	-Inhibits the growth of eosphageal squamous cells.	[193]
Formononetin	In vitro 2–6 μM	HUVEC	↑miR-375	↑ERα, Bcl-2, ↓RASD1	-Promotes cell proliferation and inhibits apoptosis.	[204]
	10–20 μM	GMCs	↑SIRT1	↑Nrf2/ARE ↓Fibronectin, ICAM-1	-Inhibits hyperglycemia-induced ROS overproduction in glomerular mesangial cells.	[224]
	40 μM	BT-549, MDA-MB-231	↓lncRNA AFAP1-AS1 ↑miR-545, miR-195	↓CDK4, Raf-1	-Inhibits proliferation, migration and invasion of TNBC cells.	[208]
	20–100 μM	SW1116, HCT116	↑miR-149	↓EphB3, cyclin D1, MMP2/9, ↓PI3K/AKT ↓STAT3	-Inhibits colon carcinoma cell proliferation and invasion.	[202]
	50–200 μM	T24	↓miR-21	↑PTEN, ↓p-Akt	-Inhibits proliferation, induces apoptosis and decreases invasiveness of bladder cancer.	[205]
	100 µM	U87MG	↓HDAC5	↓Vimentin ↑E-cadherin	-Enhances the cytotoxicity of doxorubicin in glioma cells.	[206]
	In vivo 4–8 mg/kg/day	Ovariectomized rats	↑miR-375	↑ERα, ↑Bcl-2, ↓RASD1	-Lower risk of postmenopausal breast cancer development.	[204]
	20–40 mg/kg	Diabetic type II rats	↑SIRT1 in pancreatic tissues and sciatic nerve tissue	↓MDA, ↑GSH, SOD; ↑NGF in sciatic nerve tissue.	-Reduces oxidative stress, risk of nephro-pathy and the level of triglyceride and cholesterol; -Protects from hyperglycemia induced neuronal damage.	[225,226]
	25–50 mg/kg	Diabetes mice model	↑SIRT1 in kidney tissues	↑Nrf2, ↓Fibronectin, ICAM 1	-Reduces renal fibrosis, improves renal function.	[207,224]
S-equol	In vitro 2 µM	MCF-7, MDA-MB-231	↓CpGmethylation at BRCA1, BRCA2 promoters	↑BRCA1, BRCA2	-Inhibits breast cancer cells’ proliferation.	[192]
	12.8 μM	MCF-7, MDA-MB-231	↓H3K9, H3K27, H3K4(me3) ↑H3K4ac, H4K8ac at promoter of EZH2,BRCA1, ERα, ERβ, SRC3, p300 genes	↓EZH2, ↑p300, ↑SRC3	-	[195]
	50–150 µg/mL	MCF-7	↑miR-10a-5p	↓PI3K p110α, ↓ p-Akt	-Anti-proliferative and pro-apoptotic effect.	[209]
	In vivo 10–100 mg/day	Neonatal rats	↑DNA methylation of H-ras in pancreatic cells	-	-	[227]
II. Prenylflavonoids						
6-PN 8-PN	1 μM 50–100 μM	MCF-7 SK-MEL-28	↓DNMT1 HDAC2,4,7,8 inhibition ↑H3 acetylation	↓ERα ↑P450 1A1 ↓pS6P, ↓pERK/pP90	-Activates AhR to attenuate inhibition of CYP1A1 and degradation of ERα; -Antiproliferative effects on melanoma cells.	[228,229]
Glabridin	In vitro 10 μM	MDA-MB-231 Hs-578T	↑miR-148a, ↓DNMT1 ↓DNMT3A,	↓TGFβ/SMAD2	-Inhibits the CSCs-like properties of breast cancer cells.	[167]
	10–20 μM	MDA-MB-231, Hs-578T; HepG2, Huh-7	↑miR-148a	↓Wnt/β-catenin ↓VEGF ↓SMAD2	-Attenuates angiogenes in breast cancer cells; -Inhibits the CSCs-like properties of HCC cells.	[166,230]
	In vivo 20 mg/kg/d	mouse xenograft	↑miR-148a, ↓DNMT1 ↓DNMT3A	↓TGFβ/SMAD2	-Attenuated the tumor growth, CSCs-like properties in vivo.	[167]
Icariin	1 nM	hMSCs	↓DNA methylation at ABCB1 promoter	↑ABCB1, MMP ↑P-gp protein	-Improves cellular viability, decreases oxidative stress and promotes osteogenesis of MSCs;	[231]
	10 nM	Mouse aortic ECs	↑SIRT6, ↓H3K9ac	↓NF-κB, TNF-α, ICAM-1, IL-2, IL-6	-Reduces inflammation in vitro and in vivo;	[232]
	5–10 μM	MDA-MB-231, 4T1	↑SIRT6 ↓H3K9ac	↓NF-κB p65, ↓MMP2, ↓N-cadherin ↓TNFα, ↑E-cadherin	-Suppresses migration, invasion, decreases ROS level in breast cancer cells;	[233]
	2–16 μM	IMR-90	↑SIRT6, SIRT1	↓NF-κB, ↓p-p53, p-p21, ↓Cav1	-Prevents D-gal-induced aging and cell-cycle arrest in lung fibroblast cells;	[234]
	25–50 μM	A2780	↓miR21	↑PTEN, RECK, ↓Bcl-2	-Regulates proliferation and apoptosis of ovarian cancer cells;	[235]
	In vivo 100 mg/kg	Rats	↑SIRT1, H4AcK16	↓TNF-α, IL-1β, and IL-6 ↓NF-κB(p65) phosphorylation	-Suppresses paclitaxel-induced neuroinflammation and mechanical allodynia;	[236]
	10^−2^ μM 100 mg/kg/day	FA HSPCs isolated from mice	↑SIRT6, ↓H3K9ac	↓NF-κB	-In vitro progenitor capacity; -In vivo repopulating ability of FA HSCs.	[237]
Icaritin	40 μM	CD4+T cells from SLE patients	↑H3K4me3 at Foxp3 gene ↑H3K9me3 at IL17a gene	↑Foxp3 ↓IL17a	-Reduced autoreactivity of CD4+Tcells.	[238]
III. Coumestans						
Coumestrol	In vitro 1–10 μM	Muscle cells	↑SIRT1	↑NDUFA9, SDHA, UQCRC2, COX1, PGC1, Nrf1	-Increases mitochondria number, respiratory chain proteins and mitochondrial function;	[239]
	10–50 μM	ES2	↓DNMT3A phospho	↓CK2, PCNA, ERBB2, p-AKT, p70S6K, ERK1/2, JNK1/2, p90RSK	-Preventive effects on epithelial ovarian cancer cells;	[161]
	20–40 μM	HCT116	↓H3Tr3phos	↓Haspin kinase	-Suppresses colon cancer cells’ proliferation;	[162]
	In vivo 10–100 mg/day	Neonatal rats	↑DNA methylation of H-ras	-	-	[227]
Wedelolactone	0.1–10 μM	Mino	↓EZH2, PRC2, HTM ↓H3K27me3	↓PRC2, EZH2	-Inhibition of B cell non-Hodgkin’s lymphoma cells’ proliferation;	[240]
	50 μM	HepG2, THP1, K562	↓EZH2, PRC2	↑DAB2IP, ADRB2, CDKN2A, GADD45A	-Inhibits proliferation and migration, and induces apoptosis and cell-cycle arrest of PRC2-dependent cancers.	[241]
IV. Lignans						
Arctigenin	In vitro 0.268 μM 0.5–1 μM 5 μM 20–100 μM	Rats’ neurons; SH-SY5Y; MDA-MB-231 H9C2, Rats cardiomyocyte	↑SIRT1 ↑miR-16 ↑miR-199a ↑H3K9 me3 at AP-1, Bcl-2 promoters ↑SIRT1	↓NLRP3 ↓IL-1β, ↓IL-18, ↓ASC ↓caspases-1 p20; ↓IKKα ↓IKKβ, ↓NF-κB ↓TNF-α ↓IL-6, ↑IL-10; ↓Bcl-2, ↑ phos ATF-2 ↑AMPK, ↑I-κB, ↓NFkB	-Protection against ischemic stroke, neuroprotection; -Induces anti-inflammatory, anti-apoptotic mechanisms to prevent secondary damage; -Supressed cardiomyocytes apoptosis, inflammation and oxidative stress;	[169,242,243,244]
	In vivo 100 µM/kg; 4 mg/kg/day; 20 mg/kg; 50–100 mg/kg	Rats—myocardial ischemia; xenograft mice; Rats—cerebral ischemia	↑SIRT1; ↑pho-p38; ↑SIRT1, ↑miR-96-5p ↓miR-126-5p, miR-21-5p, ↑miR-135a-5p, miR-205-5p, miR-22-3p, miR-455-5p	↑AMPK, ↑I-κB, ↓NFkB ↓Bcl-2; ↓NLRP3 IL-1β, IL-18; ↓ASC, caspases-1 p20 TIMP3, ZNF185 ↓VEGF, EGF, FGF-β, ↑Bax/Bcl-2 ratio	-Inhibition of oxidative Stress and inflammation after acute myocardial ischemia; -Protects against ischemic stroke; -Inhibited prostate tumor cell growth both in vitro and in vivo.	[169,242,244,245]
Arctiin	5 μM 10 μM	HaCaT NHDF	↑miR-125a-5p, -205-3p, -21-3p, -29b-1-5p ↓miR-3652, -494, -1246; ↑miR-602, -762, -150-3p, -4327, -584-5p, -874, -3665 ↓miR-3679-5p, -1290, -575	-Possible regulation of members of MAPK pathways and cell growth signaling pathways.	-Enhances wound healing, DNA repair in UVB-exposed keratinocytes; -Inhibits the UVB-mediated cell growth defect, apoptosis, DNA damage.	[246,247]
SGD	In vitro 50–100 µM	3T3L1	↓DNMTs, HDACs, MBD2	-	-Antioxidant effect, epigenetic modification in murine adipocytes.	[248]
	In vivo Flaxseed diet	Female mice Mice pneumonopathy	↑miR-30b, -324-5p ↓miR-382, -423; ↓miR-142-3p, -150 ↑miR-34a	Changes in mammary gland miRNome; ↓Bcl2, FGFR1	-Prevents breast cancer development during adulthood. -Antioxidant and anti-inflammatory effects.	[249,250]
Sesamin	10 µM	THP-1	↓H3/H4 acetylation at MDC promoter area, ↓CBP	↓MAPK-p38, NFkB-p65, MDC, IP-10	-Supresses allergy and asthma-related chemokines expression; -Anti-inflammatory effect.	[251]
ENL	20 μM	RWPE-1, WPE1-NA22, -NB14, -NB11, -NB26, LNCaP	↓miR-106b cluster (miR-106b, -93, -25)	↓GMNN, CDT1, MCM2, MCM 7 ↑PTEN	-Anti-proliferative effect on mid and late prostate cancers;	[252]
	50–100 µM	3T3L1	↓DNMTs, HDACs, MBD2;	-	-Antioxidant activities, downregulates epigenetic-modification-associated gene expression in murine adipocytes.	[248]
V. Stibenes						
Resveratrol	In vitro 1 μM	HaCaT	↑ SIRT1, HDAC2, ↓H4K16Ac	↑eNOS	-Accelerates wound healing repair in vitro and in vivo skin-wound models;	[253]
	5 μM	Canine-bone tissue cells MC3T3-E1	↑ SIRT1 ↓p300	↓NF-κB acetylation, IκBα phosphorylation, IKK activity kinase activity, ↑Cbfa-1	-Anti-osteoclastogenic, activates the bone-tissue cells to osteoblast and osteogenesis;	[254]
	10 μM	MDA-MB-468 A2058, M223; HCT116, SW480; HUVEC; ARPE-19	↓CpG methylation at ERα promoter; ↑SIRT1 ↑DNMT1, ↑LINE-1 methylation	↓STAT3 acethylation ↑ERα expression; ↓NF-Κb, CXCR4, MMP9; ↑eNOS acetylation	-Anti-proliferative, reduces viability and induces mesenchymal to epithelial transition phenotype in breast cancer and CRC cells; -Regulates endothelial function during oxidative stress; -Ameliorates viability and ROS production in retinal pigment epithelia cells under oxidative and inflammatory conditions;	[255,256,257,258]
	14–15 μM	MCF10A, MCF7, HCC1806, MDA-MB-157	↑CpG methylation at KCNJ4, RNF169, BCHE, DAOA ↓CpG methylation of HOXA9, KRTAP2-1, TAGAP, RUNX3; ↓CpG methylation at PTEN promoter, ↓DNMT1; ↓SIRT1, DNMT3B, ↓DNMTs activity; ↑HDACs, HATs, ↑H3Ac, H4Ac, H3K9Ac at ERα promoter	↓KCNJ4, DAOA ↑BCHE, KRTAP2-1, TAGAP; ↑PTEN, p21; ↓γ-H2AX, ↓hTERT; ↑ERα	-No cytotoxic effect on normal mammary gland cells; -Antiproliferative effect on breast cancer cells; -Induces apoptosis and cell-cycle arrest on TNBC cells.	[259,260,261,262]
	10–20 μM	MCF-7; MDA-MB-231	↓DMNT1, MBD2, H3K9me3 at BRCA1 promoter ↑H4Ac, H3K9Ac at BRCA1 promoter ↓PRMT5, EZH2, KDACs, ↑KAT2A/3B ↑global H3K9ac, H3K27ac ↓H4R3me2s, H3K27me3 at BRCA1, p53, p21 promoters	↑BRCA-1 ↓AhR and ERα at BRCA1 promoters; ↑BRCA1, p53, p21	-Attenuates dioxin carcinogenic chemicals-dependent repression of BRCA-1 and induction of DNA damage; -Inhibits breast cancer cells’ proliferation.	[263,264]
	25 μM	C2C12; A549	↑SIRT1; ↓lncAK001796	↑AMPK ↓LKB1ac, ↓PGC-1α ac ↑Nrf-1, Nrf-2, NDUFS8, SDHb, Uqcrc1, COX5b, ATP5a1 ↑BIRC5, TFDP2, CDC6 ↓ATR, CCNB1, CKS2	-Increase mitochondrial membrane potential, cellular ATP content in mouse mioblasts; -Inhibits lung cancer cells’ proliferation.	[265,266]
	20–30 μM	PANC-1, MIA PaCa-2, AsPC1; THP1	↓SIRT1, SIRT2, SIRT3; ↑miR-663, ↓miR-155	↑PTEN, p-JNK, FOXO ↓Ras, p-AKT, p-ERK, AKT kinase activity ↑caspase-3; ↓AP-1, ↑cMaf	-Induces cell-cycle arrest and apoptosis in pancreatic cancer cells; -Inhibits pancreatic tumor growth in vivo;	[267,268]
	20–50 μM	A549, BGC-823, SGC-7901 U266, LP1	↓CpG methylation at ZFP36 promoter; ↓DNMT1; ↑SIRT1; ↓lnc NEAT1	↑ZFP36, ↓CCND1, MYC, VEGFA; ↓cyclin D1, CDK4, CDK6 ↑p21, p16; ↑β-catenin cytoplasm, ↓c-Myc, MMP7, Survivin	-Inhibits migration and cell proliferation in non-small-cell lung cancer cells; -Inhibits gastric cancer cells’ proliferation and induces cell-cycle arrest; -Inhibits the tumor growth of xenografts; -Inhibits the proliferation, migration and invasion of multiple myeloma cells;	[269,270,271]
	50 µM	MCF-10A, MCF-7, MDA-MB-231 LNCaP, DU145, 22Rv1 SW480	↓CpG methylation at Nrf2, ↓miR-93; ↓HDAC activity, ↓HDAC2 at ATP2A3 promoter ↑global H3Ac, H3K27ac ↓DNMTs activity, ↓MeCP2, MBD2; ↓miRs-17-92, -106ab clusters ↓miRs-7, -17, -18b ↑miRs-150, -296-5p ↓miRs-17, -20a, -106a, -106b; ↓HDACs, NuRD complex, ↑p300; ↑miR-663, ↓miR-17, -21, -25, -92a-2	↑Nrf2; ↑ATP2A3, SERCA3; ↑PTEN; ↑PTENac, p53, ↓MTA1, PI3K-Akt; ↓TGFβ1, ↑PTEN, PDCD4, SMAD7	-Protective role against E2-induced mammary carcinogenesis; -Pro-apoptosis effect and changes in Ca^2+^ homeostasis; -Induces apoptosis and inhibits cell growth, angiogenesis and metastasis in prostate cancer cells; -Induces tumor regression in orthotopic prostate cancer xenografts; -Induces apoptosis, inhibits colon cancer cells growth.	[272,273,274,275,276,277]
	In vivo 7 ppm mixed AIN-76A diet	Pregnant female Sprague–Dawley rats	↓CpG methylation at BRCA1 promoter ↓DNMT1 at BRCA-1 promoter	↑BRCA1, AhR	-Reduces the risk of breast tumorigenesis in the offspring;	[278]
	diet with 0.4% resveratrol	Wild-type mouse	↑SIRT1	↑p-AMPK, NAD+, LKB1 acetylation	-Improves mitochondrial function and increases cellular ATP in skeletal muscle;	[265]
	5–25 mg/kg/day	Female rats bearing breast cancer	↑miRs -21, -129, -204, -489 ↓DNMT3B in tumor tissues; ↓miRs -21, -129, -204, -489 ↑DNMT3B in normal tissues		-Inhibits breast tumor formation in vivo;	[279]
	25–50 mg/kg	Mice bearing human melanoma	↓DNMT1 ↓CpG methylation at PTPN6, CDKN2A, SOCS3 promoters	↓STAT3 acetylation	-Tumor-growth inhibition;	[255]
	50 mg subcutaneous pellet/month	Female ACI rats	↓CpG methylation at Nrf2 promoter ↓miR-93	↑Nrf2, NQO1, SOD3 OGG1, FMO1, AOX1 ↓MTA1, pAkt	-Decreases tumor incidence and chemoprevention;	[272]
	extract containing resveratrol/ 1 year	Peripheral blood male with type-2 diabetes	↑miR-21, -181b, -663, -30c2 ↓miR-155, -34a	↓IL-6, CCL3, IL-1β, TNF-α ↑LRRFIP-1	-Beneficial immunomodulatory effect on hypertensive patients with type 2 diabeties.	[280]
Pterostilbene	2.5–10 μM	MCF-7 MDA-MB-231 in coculture with TAM	↑miR488	↓NF-κB, Twist1, vimentin ↑E-cadherin	-Suppresses breast EMT and/or generation of CSCs;	[173]
	5 μM	HCC1806 MDA-MB-157	↓SIRT1, ↓DNMTs activity ↑HDACs, HATs ↑H3Ac, H4Ac, H3K9Ac at ERα promoter	↓γ-H2AX, hTERT ↑ERα	-Induces apoptosis and cell-cycle arrest in breast cancer cell lines;	[261,262]
	50 μM	DU145, 22Rv1	↓miRs-17, -20a, -106a, -106b	↑PTEN ↓PI3K-Akt	-Promotes apoptosis, inhibits cell proliferation both in vitro and in vivo, and downregulates circulating tumor-derived oncomiRs in vivo.	[275]
Piceatannol	In vitro 1 μM	U937	↓miR-183	↓ADAM17, Sp1, Foxp3, TNFα/NFkB ↑ β-TrCP	-InhibitsTNF α-mediated signaling pathway in leukemia cell line;	[281]
	10 μM	THP-1, Raw264.7	↑SIRT1 ↓miR-183	↑HO-1	-Attenuates osteoclastogenesis in bone-marrow-derived macrophages;	[282]
	30 μM	RAW264.7, A2058, WM266-4, HCT116	↑miR-200a ↑miR-181a ↑miR-129	↑Nrf2 ↓NLRP3, IL-18, IL-1β, caspase1 ↑Bax, caspase 3, ↓Bcl-2	-Attenuates oxLDL-induced lipid storage by inhibiting pyroptosis in human macrophage cells; -Induces apoptosis of melanoma cells and CRC cells;	[283,284]
	In vivo 50 mg/kg/day	Renal fibrosis mice model	↓HDAC4, HDAC5	↓p38-MAPK, ECM	-Ameliorates renal fibrosis.	[285]
Resveratrol + Pterostibene	In vitro 15 μM + 5 μM	HCC1806 MDA-MB-157	↓SIRT1, ↓DNMTs, ↓Global DNA methylation ↑HDACs, HATs ↑ H3Ac, H4Ac, H3K9Ac at ERα promoter	↓γ-H2AX, hTERT, ↑ERα	-Induces apoptosis and cell-cycle arrest; -Retrieves responsiveness to E2 and 4-hydroxytamoxifen treatments in resensitized breast cancer cells.	[261,262]
	15 µM + 7 μM	MCF10A MCF10CA1h MCF10CA1a	↑CpG methylation at MAML2, GLI2 promoters; ↑DNMT3B	↓MAML2 ↓NOTCH	-Inhibition of growth of cancer cells with low and high invasive properties;	[286]
	In vivo 5–25 mg/kg/day	Rats bearing estrogen-dependent breast tumors	↓DNMT3B, ↑miR10a,−21, −129, −204, −489	-	-Delay in mammary tumor formation; -Different pattern of epigenetic changes tumor versus normal tissues;	[279]
	CSAA diet + REV 1.2 g or with PTS, 1.34 g/kg/day	Rats	DNA methylation ↓RUNX3, ↑ KCNJ4	↑RUNX3, ↓KCNJ4	-Changes theDNA methylation pattern on long-term dietary exposures.	[259]

Note: 5-mC, 5 methyl cytosine; 6-PN, 6-prenylnaringenin; 8-PN, 8-prenylnaringenin; ABCB1, ATP-binding cassette sub-family B member 1; ABCG5, ATP Binding Cassette Subfamily G Member 5; ADAM17, ADAM Metallopeptidase Domain 17; AhR, Aryl hydrocarbon receptor; AIF, Apoptosis Inducing Factor; AMPK-5’, Adenosine Monophosphate-Activated Protein Kinase; AOX1, Aldehyde Oxidase1; APC, Adenomatous Polyposis Coli; ARE, Antioxidant Responsive Element; ARID1A, AT-Rich Interaction Domain 1A; ATM, Ataxia Telangiectasia Mutated Protein; ATP5a1, ATP Synthase F1 Subunit Alpha; BARD1, BRCA1-Associated RING Domain 1; Bax, BCL2 Associated X, Apoptosis Regulator; BCHE, Butyrylcholinesterase; Bcl-2, B-cell lymphoma/leukemia protein; BIRC7, Baculoviral IAP Repeat Containing 7; BMI1, Polycomb complex protein; BRCA1,2, breast cancer type 1,2 susceptibility protein; Cav1, Caveolin 1; CCND1, cyclin D1 protein; CDK4, cyclin-dependent kinase 4; CDH1, cadherin 1; CDT1, chromatin licensing and DNA replication Factor 1; CK2, protein casein kinase 2; COX1, Cyclooxygenase-1; COX5b, Cytochrome C Oxidase Subunit 5B; CYP1A1, Cytochrome P450, family 1, subfamily A, polypeptide 1; DAOA, D-amino acid oxidase activator; DAPK1, Death Associated Protein Kinase 1; Dkk2, Dickkopf WNT Signaling Pathway Inhibitor 2; DNMT1, 3A, 3B DNA Methyltransferase 1, 3A, 3B; EED, Embryonic Ectoderm Development; EGFR, Epidermal Growth Factor Receptor; ERK1/2 extracellular signal-regulated protein kinase ½; EP300/p300 E1A Binding Protein P300; EPHB2 Ephrin type-B receptor 2; ERα, ERβ Estrogen receptor alpha, beta; ERBB-4, Erb-B2 Receptor Tyrosine Kinase 4; ESR1, 2 Estrogen receptor 1, 2; EZH2 Enhancer of Zeste 2 Polycomb Repressive Complex 2 Subunit; FHIT (Fragile Histidine Triad Diadenosine Triphosphatase; FMO1 Flavin Containing Dimethylaniline Monoxygenase 1; FOXO3 Forkhead Box O3; GSH Glutathione; GSTP1 Glutathione S-Transferase Pi 1; HES1 Hes Family BHLH Transcription Factor 1; H3K9me3, Histone 3 lysine 9 trimethylation; H3K27me3 Histone 3 lysine 9 trimethylation; H3K4ac Histone H3 acetylated at lysine 4; H4K8ac Histone H4 acetylated at lysine 8; HDAC1, 2, 5 Histone Deacetylase 1, 2, 5; HOXA5, A9, A11, B1 Homeobox proteins; HO-1 heme oxygenase-1; hTERT Telomerase reverse transcriptase; ICAM 1 Intercellular Adhesion Molecule 1; IL-1β2,6, Interleukin-1β,2, 6; IX, isoxanthohumol; JAK/STAT, Janus kinase/signal transducer and activator of transcription; JNK1/2, c-Jun N-terminal kinases ½; KAT2A/3B, (Lysine Acetyltransferase 2A/3B); KCNJ4, Potassium Inwardly Rectifying Channel Subfamily J Member 4; KDACs, Lysine (K) deacetylases; KDM4B, Lysine Demethylase 4B; KRTAP2-1, keratin-associated protein 2-1; LSD1, Lysine-specific histone demethylase 1A; LKB1, Liver Kinase B1; MAD1L1 mitotic arrest deficient 1–like 1; MBD2, Methyl-CpG-binding domain protein 2; MCM7, Minichromosome Maintenance Complex Component 7; MeCP2, Methyl-CpG Binding Protein 2; MGMT O-6-Methylguanine-DNA Methyltransferase; MMP2,7,9 matrix metallopeptidase 2,7,9; NF-Κb Nuclear factor-kappa B; NGF Nerve growth factor; Nrf1 Nuclear Respiratory Factor 1; Nrf2 Nuclear Factor Erythroid 2–like 2; NDUFA9 NADH:Ubiquinone Oxidoreductase Subunit A9; NDUFS8 NADH:Ubiquinone Oxidoreductase Core Subunit S8; NOTCH Notch Receptor; *O*-DMA *O*-demethylangolensin; OGG1 oxidative DNA damage repair gene; p16 multiple tumor-suppressor 1 or cyclin-dependent kinase inhibitor 2A; p27 Cyclin-dependent kinase inhibitor 1B;p53/ TP53 transformation-related protein 53; p65 nuclear factor NF-kappa-B; p70S6K phosphorylated ribosomal protein S6 kinase; p90RSK phosphorylated Serine/threonine-protein kinase; P450 1A1 Cytochrome P450, Family 1, Subfamily A, Polypeptide 1; p-AKT phosphorylated Protein kinase B; PCNA Proliferating Cell Nuclear Antigen; pS6P phosphorylated (Ser240/244) S6 ribosomal protein; pERK PKR-like ER kinase;PI3K p110α phosphatidylinositol 3-kinase P110 Subunit Alpha;PcG polycomb-group proteins;P-gp P-glycoprotein 1; PGC-1α peroxisome proliferator-activated receptor gamma coactivator 1-α; PRC2 Polycomb repressive complex 2; PRMT5 Protein Arginine Methyltransferase 5; PTEN Phosphatase and Tensin Homolog; RAC1 Rac Family Small GTPase 1; RAD23B UV Excision Repair Protein RAD23 Homolog B; Raf1 Proto-Oncogene Serine/Threonine Protein Kinase; RASD1 Ras Related Dexamethasone Induced 1; RARβ2 retinoic acid receptor beta2; RECK Reversion Inducing Cysteine Rich Protein with Kazal Motifs; REPS2 RALBP1-associated Eps domain containing 2; RNF169 ring finger protein 169; RUNX3 runt-related transcription factor 3; SDHb Succinate Dehydrogenase Complex Iron Sulfur Subunit B; SFRP1 Secreted Frizzled Related Protein 1; Smad 2,4 SMAD Family Member 2,4; SDHA Succinate Dehydrogenase Complex Flavoprotein Subunit A; SIRT1,6, silent mating type information regulation 2 homolog 1,6; SLUG/SNAIL, Snail Family Transcriptional Repressor 2; SMARCB1, SWI/SNF-Related Matrix-Associated Protein; SOCS1, Suppressor of Cytokine Signaling 1; SOD, Superoxide Dismutase; SOX7, SRY-Box Transcription Factor 7; SRC3, Nuclear Receptor Coactivator 3; TAGAP, T-cell activation RhoGTPase-activating protein; TGFβ, Transforming Growth Factor-Beta; TGFBIII, Transforming Growth Factor Beta Induced; TNFα, Tumor Necrosis Factor; TRAF7, TNF Receptor Associated Factor 7; Uqcrc1,2, Ubiquinol-Cytochrome C Reductase Core Protein 1,2; VEGFA, Vascular Endothelial Growth Factor A; ZFP36, ZFP36 Ring Finger Protein; ZO-1, Tight junction protein-1; XN, Xanthohumol; XRCC2, X-Ray Repair Cross Complementing 2. *Cell lines*: 22Rv1 human prostate carcinoma epithelial; 3T3L1 mouse fibroblast-cell line capable of differentiating into adipocytes; 4T1 mouse breast cancer epithelial, resistant to 6-thioguanine; 786-O human renal adenocarcinoma; A2058 human melanoma; A2780 human ovarian carcinoma; A-498 human renal carcinoma; A549 human adenocarcinoma alveolar basal epithelial cells; ACHN human papillary renal cell carcinoma; ARCaP-E, ARCaP-M human prostate cancer cells; ARPE-19, spontaneously arising retinal pigment epithelia (RPE) cells; AsPC-1 human pancreatic adenocarcinoma; BGC-823 human-papillomavirus-related endocervical adenocarcinoma; BT-474 human invasive ductal carcinoma; BT-549 human ductal carcinoma; Caki-2 human renal carcinoma; C2C12 immortalized mouse myoblast; DU145-human prostate cancer (AR+); ES2 human ovarian carcinoma; Ecs mouse aortic endothelial cells; FA HSPCs human Fanconi anemia hematopoietic stem cells; GMCs, human glomerular mesangial cells; H9C2 rat myoblast cells; HaCaT, human aneuploid immortal keratinocyte; HCC1806 human breast cancer (ER-, PR-); HCT116 human colorectal carcinoma; HeLa human cervical cancer; HepG2 human hepatocellular carcinoma; HMECs normal human mammary epithelial; hMSCs, Human Mesenchymal Stem Cells; Hs578T human breast carcinoma (ER-); HT29 human recto-sigmoid adenocarcinoma; Huh-7 human hepatocellular carcinoma; HUVEC, Human Umbilical Vein Endothelial Cells; IMR-90 human embryonic lung fibroblasts; K562 human erythroleukemia; KYSE-510 human eosphageal squamous cell carcinoma; LAPC-4 human prostate cancer (AR+, PSA+); LNCaP, Lymph Node Carcinoma of the Prostate cell line (AR+, ER+); LP-1 human myeloma; M223 human etastatic melanoma; MC3T3-E1 mouse pre-osteoblastic cells; MCF7 human breast adenocarcinoma (ER+); MCF10Ahuman breast non-tumorigenic epithelial cells; MCF10CA1h human malignant breast cells; MCF10CA1a human pre-neoplastic mammary gland cells; MDA-MB-157, MDA-MB-231, MDA-MB-435 and MDA-MB-468 human metastatic breast carcinoma cells (ER-, PR-, Her2-); MGC-803 human gastric cancer cells; Mino human mantle cell lymphoma; NHDF human dermal fibroblast cells; MIA PaCa-2 human pancreatic carcinoma; PANC-1 human pancreatic epithelioid carcinoma; PC-3-human prostate adenocarcinoma; RAW264.7 murine macrophage cells; RWPE-1 human immortalized, normal prostate cells; SGC-7901 human gastric cancer cells; SH human precancerous breast cells expressing hTERT; SHR human completely transformed, breast cancer cells; SH-SY5Yhuman neuroblastoma; SiHa human cervix squamous carcinoma; SKBR3 human breast adenocarcinoma; SK-MEL-28 human melanoma; SW1116 human colorectal adenocarcinoma; SW480 human colon adenocarcinoma; SW620 human metastatic colon adenocarcinoma; T24 human bladder cancer; T-47D human breast carcinoma; TAM tumor-associated macrophages; THP-1 human monocytic leukemia; UACC-3199 human mammary gland ductal carcinoma c (ERα-); U-87MG human glioblastoma; U266 human B lymphocyte myeloma; U937 human monocyte histiocytic lymphoma; WM266-4 human metastatic melanoma; WPE1–human prostate cells with different grade of malignancy, increasing from NA22, NB11, NB14, to NB26.

## Supplementary Materials

The following is available online at https://www.mdpi.com/article/10.3390/antiox10121893/s1, Table S1: Chemical Abstracts Services numbers of Phytoestrogens.
