# Supplementary material for: Dietary Phytoestrogens and Their Metabolites as Epigenetic Modulators with Impact on Human Health
Source: Antioxidants (Basel). 2021 Nov 26;10(12):1893. doi: 10.3390/antiox10121893 (PMC8750933; doi:10.3390/antiox10121893)
Supplement: Supplementary file 1 [file antioxidants-10-01893-s001.zip › antioxidants-1456052-supplementary.pdf]

antioxidants

MDPI

Table S1. Chemical Abstracts Services numbers of Phytoestrogens.

Phytoestrogens	CAS number
**I. Isoflavones**	
Biochanin A	491-80-5
Daidzein	486-66-8
Daidzin	552-66-9
Dihydrodaidzein	17238-05-0
Dihydrogenistein	21554-71-2
Formononetin	485-72-3
Genistein	529-59-9
Genistin	529-59-9
Glycitein	40957-83-3
Glycitin	40246-10-4
O-Desmethylangolensin (O-DMA)	21255-69-6
S-equol	531-95-3
**II. Prenylflavonoids**	
6-Prenylnaringenin (6-PN)	68682-01-9
8-Prenylnaringenin (8-PN)	68682-02-0
Desmethylicaritin	28610-31-3
Desmethylxanthohumol (DMX)	115063-39-3
Glabridin	59870-68-7
Icariin	489-32-7
Icariside I	56725-99-6
Icariside II	113558-15-9
Icaritin	118525-40-9
Isoxanthohumol (IX)	521-48-2
Xanthohumol (XN)	6754-58-1
**III. Coumestans**	
4′-methoxycoumestrol	1690-62-6
Coumestrol	479-13-0
Repensol	33280-69-2
Wedelolactone	524-12-9
**IV. Lignans**	
Arctigenin	7770-78-7
Arctiin	20362-31-6
Enterodiol (END)	80226-00-2
Enterolactone (ENL)	78473-71-9
Isolariciresinol	548-29-8
Lariciresinol (LARI)	27003-73-2
Matairesinol (MAT)	580-72-3
Medioresinol	40957-99-1
Pinoresinol (PINO)	487-36-5
Secoisolariciresinol (SECO)	29388-59-8
Secoisolariciresinol diglucoside (SDG)	148244-82-0
Sesamin	607-80-7
Sesamolin	526-07-8
**V. Stilbenes**	
3,4′-dihydroxybibenzil (Lunularin)	37116-80-6
Cis-piceid	148766-36-3

Dihydroresveratrol (DHR)	58436-28-5
Isorhapontigenin	32507-66-7
Piceatannol	10083-24-6
Pterostilbene	537-42-8
Resveratrol	501-36-0
Trans-piceid	27208-80-6

CAS: Chemical Abstracts Services
